# A supervised Bayesian factor model for the identification of multi-omics signatures

**DOI:** 10.1093/bioinformatics/btae202

**Published:** 2024-04-11

**Authors:** Jeremy P Gygi, Anna Konstorum, Shrikant Pawar, Edel Aron, Steven H Kleinstein, Leying Guan

**Affiliations:** Program in Computational Biology & Bioinformatics, Yale University, New Haven, CT 06520, United States; Department of Pathology, Yale School of Medicine, New Haven, CT 06520, United States; Department of Genetics, Yale Center for Genomic Analysis (YCGA), Yale School of Medicine, New Haven, CT 06520, United States; Program in Computational Biology & Bioinformatics, Yale University, New Haven, CT 06520, United States; Program in Computational Biology & Bioinformatics, Yale University, New Haven, CT 06520, United States; Department of Pathology, Yale School of Medicine, New Haven, CT 06520, United States; Department of Immunobiology, Yale School of Medicine, New Haven, CT 06520, United States; Department of Biostatistics, Yale School of Public Health, New Haven, CT 06520, United States

## Abstract

**Motivation:**

Predictive biological signatures provide utility as biomarkers for disease diagnosis and prognosis, as well as prediction of responses to vaccination or therapy. These signatures are identified from high-throughput profiling assays through a combination of dimensionality reduction and machine learning techniques. The genes, proteins, metabolites, and other biological analytes that compose signatures also generate hypotheses on the underlying mechanisms driving biological responses, thus improving biological understanding. Dimensionality reduction is a critical step in signature discovery to address the large number of analytes in omics datasets, especially for multi-omics profiling studies with tens of thousands of measurements. Latent factor models, which can account for the structural heterogeneity across diverse assays, effectively integrate multi-omics data and reduce dimensionality to a small number of factors that capture correlations and associations among measurements. These factors provide biologically interpretable features for predictive modeling. However, multi-omics integration and predictive modeling are generally performed independently in sequential steps, leading to suboptimal factor construction. Combining these steps can yield better multi-omics signatures that are more predictive while still being biologically meaningful.

**Results:**

We developed a supervised variational Bayesian factor model that extracts multi-omics signatures from high-throughput profiling datasets that can span multiple data types. Signature-based multiPle-omics intEgration via lAtent factoRs (SPEAR) adaptively determines factor rank, emphasis on factor structure, data relevance and feature sparsity. The method improves the reconstruction of underlying factors in synthetic examples and prediction accuracy of coronavirus disease 2019 severity and breast cancer tumor subtypes.

**Availability and implementation:**

SPEAR is a publicly available R-package hosted at https://bitbucket.org/kleinstein/SPEAR.

## 1 Introduction

Biological signatures, composed of subsets of predictive analytes, serve as valuable biomarkers for disease diagnosis and prognosis (Ramilo *et al.* 2007, Yu *et al.* 2019, Bodkin *et al.* 2022, Chawla *et al.* 2022), as well as the prediction of responses to vaccinations and therapies (Fourati *et al.* 2022, Hagan *et al.* 2022). In addition to being predictive, signatures offer interpretable insight into the underlying mechanisms that drive disease, further adding to their value to the scientific community. By leveraging associations between different types of analytes, multi-omics signatures offer the possibility of improved performance and greater insights.

The identification of predictive biological signatures continues to be a prominent area of focus in biomedical research. For example, Fourati *et al.* (2022) and Hagan *et al.* (2022) identified pre- and post-vaccination gene signatures predictive of antibody responses across 13 different vaccines by random forest classification and logistic regression, respectively. Walker *et al.* (2023) identified potential protein biomarkers of dementia via Cox proportional hazards regression modeling. Signatures can also provide mechanistic insight into underlying biological processes that affect the response of interest. Nakaya *et al.* (2011) found a gene signature predictive of seasonal TIV vaccination response that included TLR5, known to sense bacterial flagellin. High association between TLR5 and TIV vaccination prompted the study and hypothesis that other ligands for TLR5, such as microbiota, are involved in influencing adaptive immunity to vaccination. These studies, like most studies, identify signatures that are derived from a single biological assay.

Recent advancements in high-throughput technologies have enabled the simultaneous collection of data from multiple assays (or ‘omics’) from the same sample (Bhattacharya *et al.* 2014). These multi-omics datasets have the potential to yield multi-omics signatures that integrate biological signals across different modalities to predict a response of interest. While the dimensionality of multi-omics datasets is high, often only a small number of key underlying factors are used to capture the major data variation from the different assays. For example, activation of the interferon signaling pathway can drive hundreds of genes (Bolen *et al.* 2014), proteins (Lazear *et al.* 2019), and metabolites (Banoth and Cassel 2018) to vary together across samples. One option to identify these major sources of variation is through the employment of latent factor models (Cantini *et al.* 2021), which construct low-dimensional factors from groups of biological analytes via dimensionality reduction. Unsupervised dimensionality reduction techniques for discovering factors are widely used in practice, including both non-probabilistic approaches such as principal component analysis, single and multi-block canonical correlation analysis (Tenenhaus and Tenenhaus 2011, Tenenhaus *et al.* 2014) as well as probabilistic approaches based on Bayesian factor models including Multi-omic Factor Analysis (MOFA) (Argelaguet *et al.* 2020) and iClusterBayes (Mo *et al.* 2018).

Conventionally, when identifying predictive signatures, the process of data integration and predictive modeling are performed separately, with multi-omics factors being constructed before subsequent association with a response of interest. Combining these steps can lead to better multi-omics signatures that are more predictive while still being biologically interpretable (Li *et al.* 2012, Singh *et al.* 2019). To accomplish this, we present Signature-based multiPle-omics intEgration via lAtent factoRs (SPEAR). The SPEAR model employs a probabilistic Bayesian framework to jointly model multi-omics data with response(s) of interest, emphasizing the construction of predictive multi-omics factors. SPEAR estimates analyte significance per factor, extracting the top contributing analytes as a signature. In addition, the SPEAR model is amenable to various types of responses in both regression and classification tasks, permitting both continuous responses such as antibody titer and gene expression values, as well as categorical responses like disease subtypes. We demonstrate SPEAR’s advantages under simulated settings and validate SPEAR’s improved performance on real public multi-omics datasets for breast cancer and SARS-CoV-2 (COVID-19) through multi-class area under the receiver operating characteristic (AUROC) testing and balanced misclassification errors.

## 2 Materials and methods

**Figure 1.**
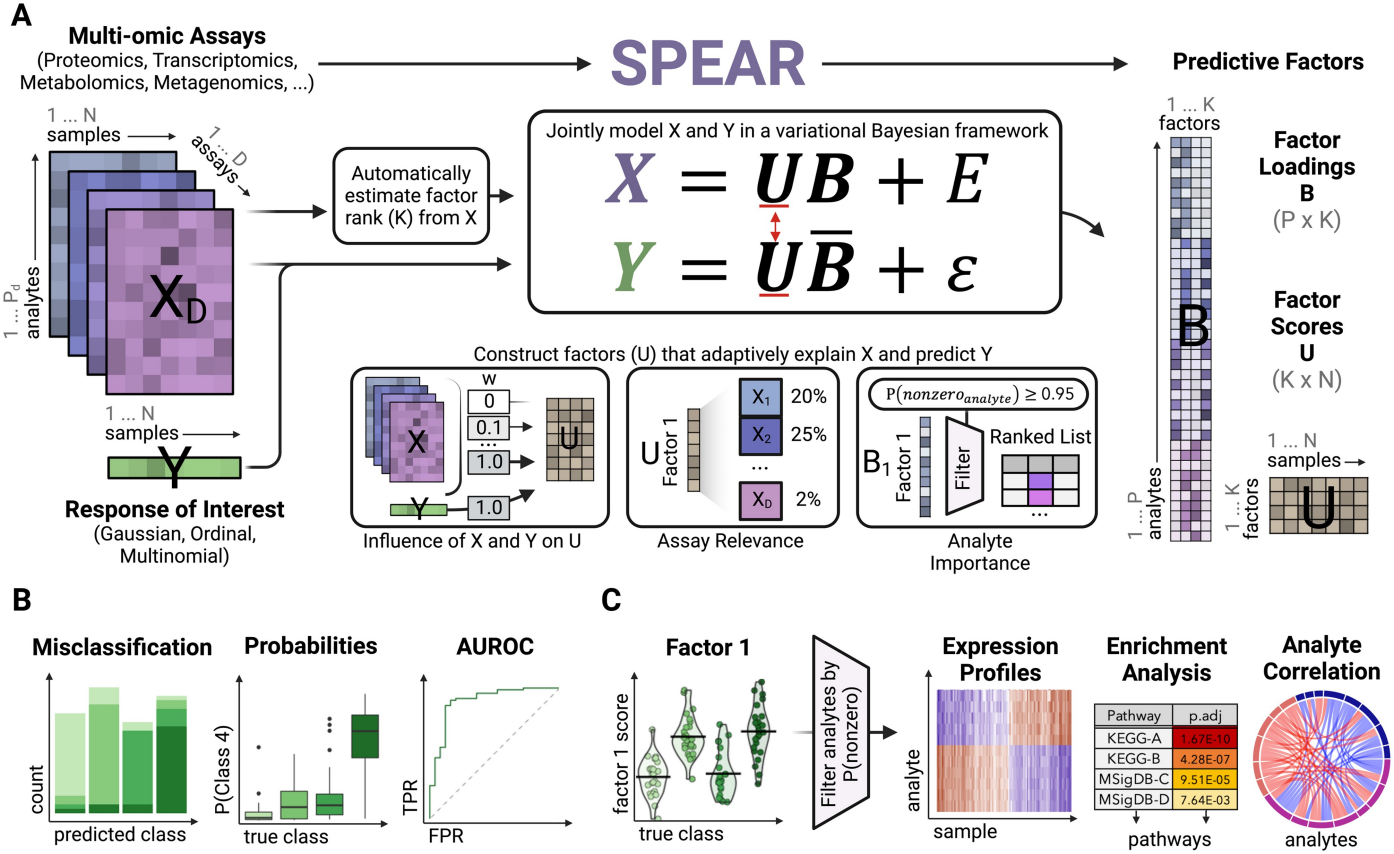
SPEAR workflow overview: (A) SPEAR takes multi-omics data (X) taken from the same N samples, as well as a response of interest (Y). SPEAR supports Gaussian, ordinal, and multinomial types of responses. From these inputs, the algorithm first automatically estimates the minimum number of factors to use in the SPEAR model. X and Y are then jointly modeled in a variational Bayesian framework to adaptively construct factor loadings (B) and scores (U) that explain variance of X and are predictive of Y (reflected in $\bar{B}$). (B) SPEAR factors are used to predict Y and provide probabilities of class assignment for ordinal and multinomial responses. (C) Downstream biological interpretation of factors is facilitated via automatic feature selection, expression profile analysis, enrichment analysis, and analyte correlation

SPEAR identifies multi-omics signatures through the construction of predictive factors via dimensionality reduction. SPEAR jointly models both high-dimensional multi-omics assays (X) and a low-dimensional response of interest (Y) and approximates posteriors of the factor loadings (β) and factor scores (U) using the variational Bayes inference (Fig. 1 and Supplementary Methods B2).

Let X be an N×p high-dimensional matrix representing the concatenation of multiple multi-omics assays for N samples and p features. We assume that X is driven by underlying low dimensional factors via linear modeling, coupled with unstructured Gaussian noise (E):

(1) $$X_{j}=UB_{j}\;+\;E_{j},\;\mathrm{for}\;\mathrm{all}\;j=1,\ldots ,\;p.$$

The goal of latent factor model analysis is to decompose X into latent factors (U) and factor loadings ($B_{j}$ for all j=1, …, p). This has been previously accomplished by finding the Bayesian posteriors of U and B in the probability model (Argelaguet *et al.* 2020) but has not yet been extended to work with a response of interest. Let Y be a length N vector representing a univariate Gaussian response of interest (see Supplementary Methods C for extensions to ordinal, multinomial, binomial, and multiple responses). Like X, let Y also be constructed by factors via linear modeling:

(2) $$Y\;=\;U\bar{B}\;+\;\varepsilon ,$$

where $\bar{B}\;$is a vector of response coefficients used to construct *Y*. Factors that are most influential for the prediction of Y are indicated by larger magnitudes in corresponding $\bar{B}.$

SPEAR prioritizes the estimation of predictive factors by jointly modeling X and Y and considering a weighted likelihood model where the weight parameter (w) indicates the emphasis on exploring existing factor structure in X:

(3) $$P^{w}\left(X,Y|U\right)=P^{w}\left(\left.X\right|U\right)\;\times \;P\left(\left.Y\right|U\right),$$

where $P^{w}\left(\left.X\right|U\right)$ is the weighted likelihood of X given U and $P\left(\left.Y\right|U\right)$ is the likelihood of Y given U. When w is large (w ≥ 1), SPEAR will emphasize the construction of factors that explain the structure of the multi-omics assays (X). Intermediary weights (1 > w > 0) correspond to a gradual shift in emphasis of predicting the response (Y) over explaining assay variance. When w is smallest (w≈0), SPEAR will forgo any attempt to reconstruct factors from the data and focus entirely on optimizing the prediction of the response. SPEAR automatically selects the weight w via cross-validation to balance both a high-prediction accuracy and explainable factors (when these are supported by the data) (Section 2 and Supplementary Methods A2).

## 3 Results

### 3.1 SPEAR improves prediction of synthetic responses from simulated multi-omics data

We first evaluated the ability of SPEAR to predict a Gaussian response on simulated data. In the simulation, five predetermined factor signals (U) were used to construct four multi-omics assays (X), each with 500 analytes (for a total of 2,000 simulated features), for 500 training and 2,000 test samples. The first two simulated factors were assigned to be the multi-omics signatures and were used to construct a Gaussian response vector (Y) for both the training and test groups, whereas the remaining three simulated factors were only used to construct X. This procedure was repeated across a gradient of signal-to-noise ratios (low signal, moderate signal, and high signal), with 10 independent iterations for each ratio (Supplementary Methods D).

**Figure 2.**
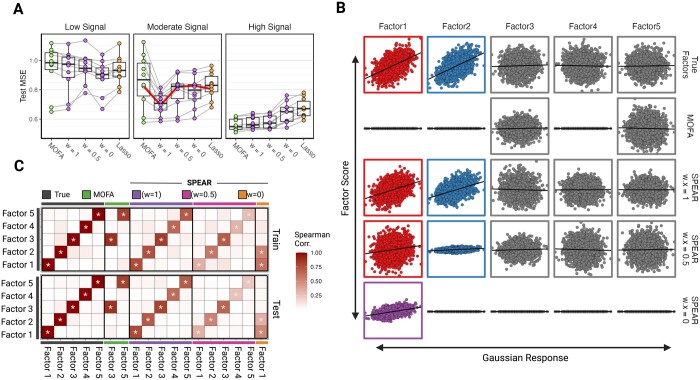
Gaussian simulation results. (A) Boxplots of mean-squared errors of the models on the testing data. MSE results for each simulated iteration are connected. Results are shown for varying signal-to-noise ratios, including low, moderate, and high signals. (B) Scatterplots of various factor scores (*y*-axis) against the true Gaussian response (*x*-axis) of the moderate signal test data. Color is applied to factor scores found to be correlated with true factors 1 (red) and 2 (blue) with true factors 3–5 designated as grey. (C) Correlation matrix showing the Spearman correlation between each derived factor of the true factors for both the training and testing data of the moderate signal simulated dataset. Significant correlations are denoted with *P ≤ 0.001

We trained SPEAR across a gradient of values for weight w to demonstrate the effect of w on SPEAR’s performance. As a comparison, we also employed a two-step MOFA-based model that performs Lasso regression (Tibshirani 1996) using factors derived from MOFA (denoted as MOFA in the following) as well as a vanilla Lasso regression model using concatenated features in lieu of factors (denoted as Lasso). Model comparison was measured by calculating the mean squared error (MSE) of the test data as well as Pearson correlation testing of the constructed factors against both Y (Fig. 2B) and U (Fig. 2C).

In scenarios with moderate signal-to-noise, SPEAR with w=1 significantly outperformed MOFA (Fig. 2A). Correlation analyses of the factor scores for one iteration (denoted by the red line in Fig. 2A) confirmed that SPEAR with w=1 correctly identified all five simulated factors, whereas MOFA only identified two of the uncorrelated factors (Factor 3 and Factor 5) likely due to the lack of supervision (Fig. 2C), which was confirmed across all 10 iterations (Supplementary Fig. S2). As expected, SPEAR with w=0 condensed all predictive signals into a single factor, as evidenced by its correlation with the first two simulated factors. Overall, SPEAR with higher weights achieved the best predictive performance due to better reconstruction of the multiple underlying simulated factors.

Finally, we repeated the above protocol to test the ability of SPEAR for predicting various non-Gaussian responses (Supplementary Methods A1). Simulated factor construction followed the same protocol above, with only the first two of five factors containing nonlinear signals that were predictive of the response (Supplementary Figs S3A and B and 4A and B). When the response type was modeled properly (e.g. Gaussian, multinomial, ordinal), SPEAR achieved the best performance via balanced misclassification errors (Supplementary Figs 3C and D and 4C and D).

### 3.2 SPEAR improves prediction of breast cancer tumor subtypes and COVID-19 severity

To test whether SPEAR could achieve competitive performance in real data, we applied SPEAR to two publicly available multi-omics datasets: a breast cancer dataset of tumor samples by *Singh et al.* (Singh *et al.* 2019) with the goal of tumor subtype prediction, and a SARS-CoV-2 patient dataset of blood samples by Su *et al.* (2020) with the goal of predicting disease severity. We refer to these datasets as TCGA-BC (The Cancer Genome Atlas (Cancer Genome Atlas Network 2012)—Breast Cancer) and COVID-19, respectively. The TCGA-BC dataset is composed of 989 biopsy samples each associated with RNA-Seq, miRNA, and methylation probe data from primary solid breast cancer tumors that have been annotated according to the PAM50 subtype signature into one of four subtypes: Luminal A (LumA), Luminal B (LumB), HER2-enriched (HER2) and Basal-like (Basal) (Parker *et al.* 2009). The COVID-19 dataset contains plasma protein and metabolite compositions for 254 SARS-CoV-2 positive patient samples and 124 matched healthy subject samples. Each subject is associated with a severity score based on the World Health Organization (WHO) ordinal severity score (Rubio-Rivas *et al.* 2022), binned into four ordinal classes (healthy, mild, moderate, and severe).

We applied SPEAR, Lasso, MOFA, and DIABLO to predict the response from each multi-omics dataset. Datasets were preprocessed and split into training and testing cohorts (see Section 2). Classification performance was evaluated via the balanced misclassification error rate and the AUROC curves. AUROC significance was calculated via a bootstrapping procedure and compared via DeLong’s test (DeLong *et al.* 1988).

**Figure 3.**
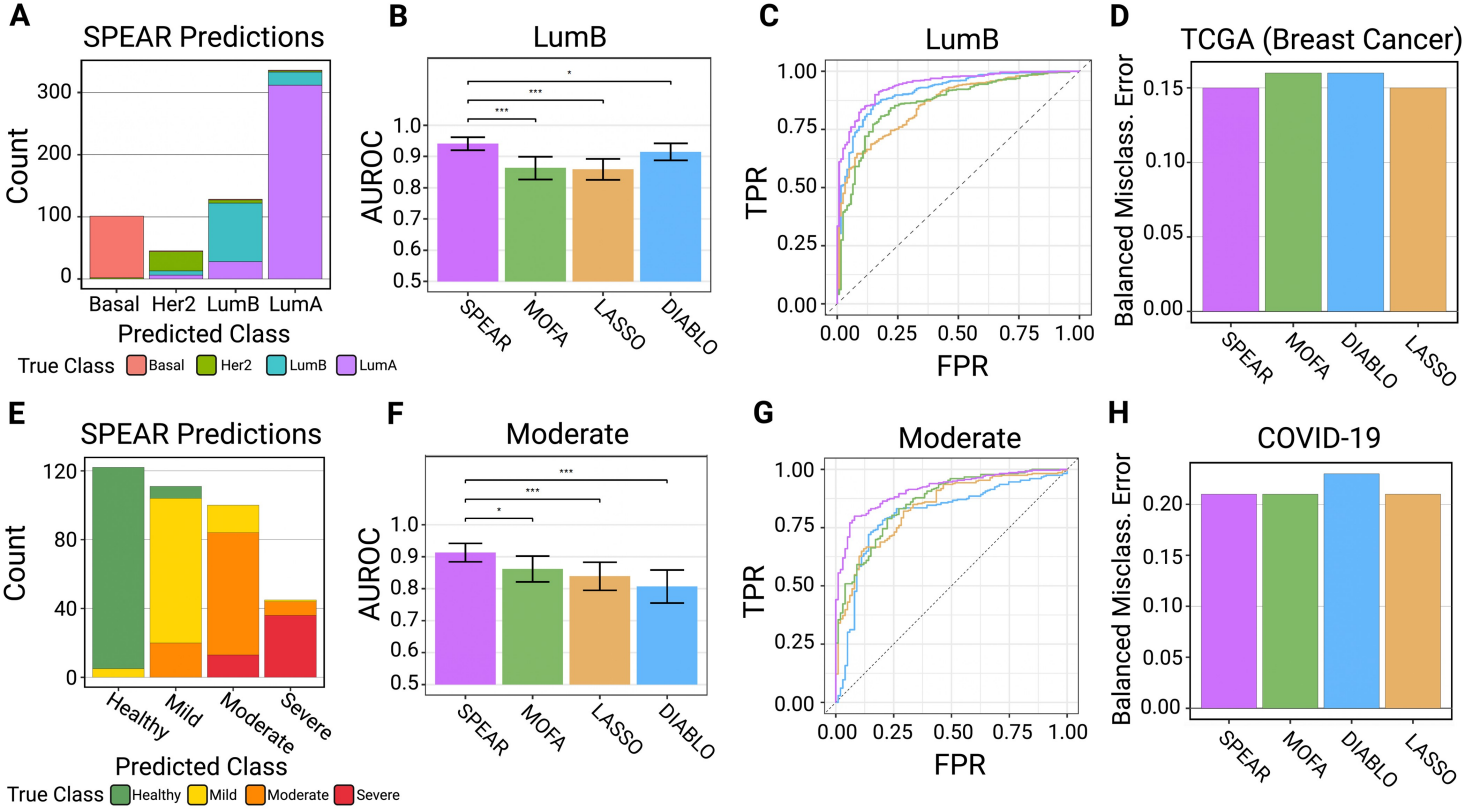
TCGA-BC Tumor Subtype and COVID-19 Severity Prediction Results. (A, E) Test sample class predictions of the SPEAR model, colored by true class. (B, F) Multi-class AUROC statistics for each model for the LumB and Moderate classes. Error bars show the 95% confidence interval found via 2,000 stratified bootstrapping replicates. Significance testing is denoted as *P ≤ 0.05), **P ≤ 0.005, and ***P ≤ 0.0005. (C) AUROC plot for all models predicting LumB subtype. (G) AUROC plot for all models predicting the moderate severity class. (D, H) Balanced misclassification errors of SPEAR, MOFA, DIABLO, and Lasso on test samples from the (D) TCGA (Breast Cancer) dataset and (H) COVID-19 dataset

The advantage of SPEAR was clear when looking at the multi-class AUROC, measuring a classifier’s ability to discriminate each class individually across a gradient of threshold values. SPEAR showed higher AUROC in discriminating between all classes and was significantly better for multiple classes: SPEAR was significantly better at predicting both the LumB subtypes from the TCGA-BC dataset (Fig. 3B and C), and moderate SARS-CoV-2 severity from the COVID-19 dataset (Fig. 3 F and G) compared to all other models via AUROC comparison testing (LumB—MOFA: *P*.adj = 7.8e-09, Lasso: *P*.adj = 2.1e-07, DIABLO: *P*.adj = 1.9e-02; Moderate—MOFA: *P*.adj = 1.4e-02, Lasso: *P*.adj = 1.9e-04, DIABLO: *P*.adj = 1.5e-05). Upon investigation, the improved predictive performance of SPEAR on these classes was not due to a single factor but was rather achieved through combining information from multiple multi-omics factors (Supplementary Figs 5 and 6). The balanced misclassification errors of SPEAR (0.15, 0.21) were comparable to those of Lasso (0.15, 0.21), MOFA (0.16, 0.21), and DIABLO (0.16, 0.23) for the TCGA-BC dataset and COVID-19 dataset respectively (Fig. 3A, D, E, H). Overall, our results demonstrate that SPEAR outperforms current state-of-the-art methods for predicting a response using multi-omics data.

It was notable that the balanced misclassification error of SPEAR was comparable with other methods even though SPEAR does not optimize purely for predictive performance. Rather, SPEAR favors multi-omics assay influence in the construction of predictive factors by choosing the largest w whose mean cross-validated error falls within one standard deviation of the overall minimum cross-validated error. If we instead choose the w that minimizes the cross-validated error (denoted as SPEAR *min*), the balanced misclassification error is better than all other models on both the TCGA-BC and COVID-19 datasets (0.13, 0.19) (Supplementary Fig. S7A and D). Similarly, this approach achieves the best AUROC values for all classes (Supplementary Fig. S7C and F). The SPEAR min model showed only a slight improvement in classification accuracy but chose considerably lower w values (w = 0.5 for TCGA-BC and w = 0.01 for COVID-19) than the default SPEAR model (denoted as SPEAR sd when needed for clarity) (w = 2.0 for both datasets) (Supplementary Fig. S8). Although we recommend and used the default SPEAR model to enhance the downstream interpretation, SPEAR *min* can be used in cases where prediction is the key objective regardless of multi-omics assay influence in factor construction.

### 3.3 SPEAR identifies steroid response pathways associated with TCGA PAM50 subtypes

**Figure 4.**
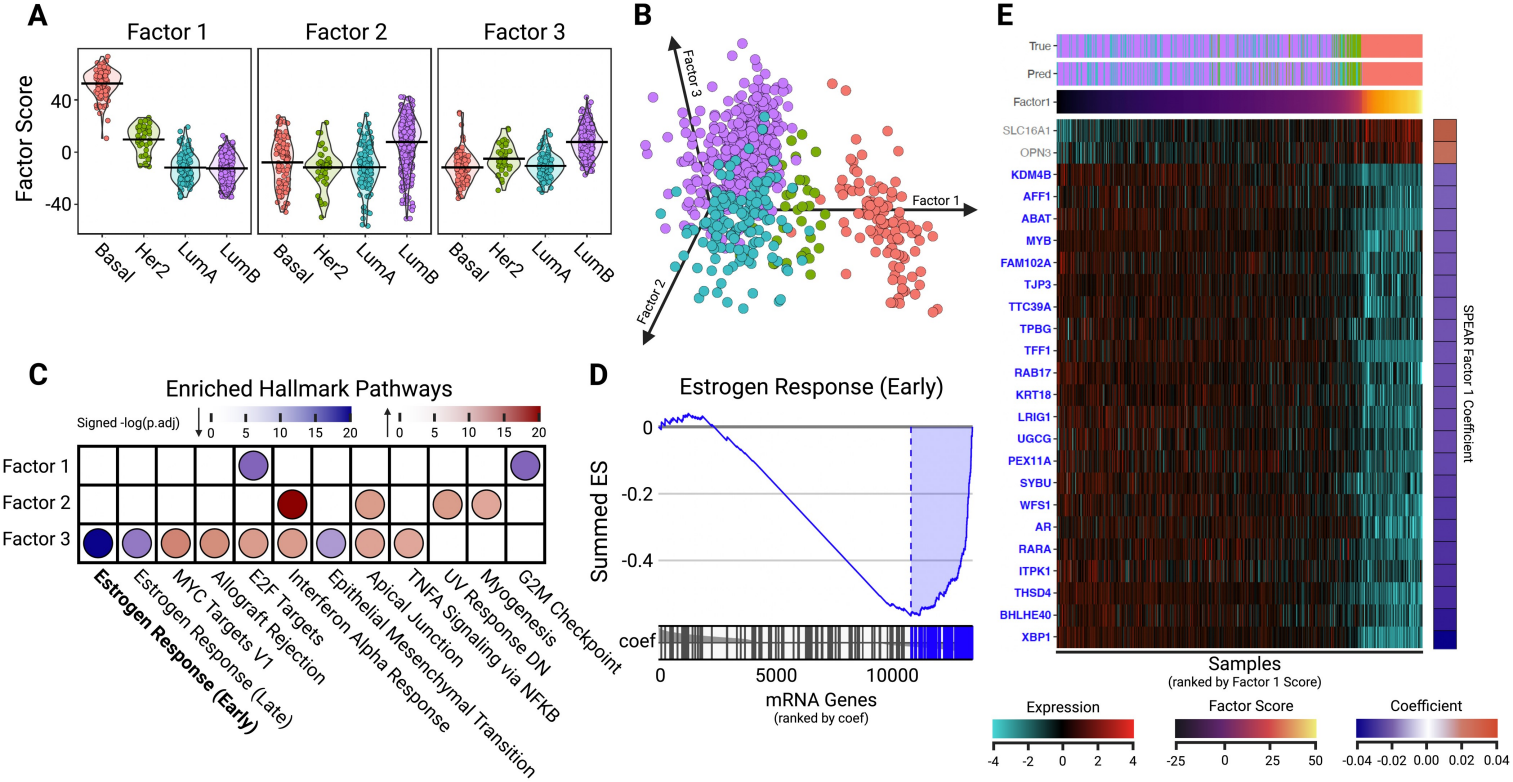
Downstream TCGA-BC Analysis. (A) Grouped violin plot of Factors 1–3 scores (*y*-axis) and tumor subtype (*x*-axis), with group means marked with a line. (B) 3D scatter plot, embedding samples by Factors 1, 2, and 3 scores. Samples are colored by tumor subtype. (C) Dotplot of GSEA results on mRNA features for Factors 1–3. Points are shaded by −log(*P*.adjusted) with color representing enrichment direction. (D) GSEA plot for Estrogen Response (Early) Hallmark pathway for SPEAR Factor 1. mRNA genes are ranked by their assigned projection coefficient from SPEAR Factor 1. (E) Heatmap showing normalized expressions for the top 24 mRNA genes involved in the Estrogen Response (Early) Hallmark pathway. mRNA genes were selected with a factor loading (projection coefficient) magnitude ≥0.02. Samples were ranked by Factor 1 score (*x*-axis) and genes were ranked by projection coefficient (*y*-axis). Also shown are corresponding true tumor subtypes (True) and SPEAR-predicted tumor subtypes (Pred)

Several factors returned by SPEAR were associated with different biological pathways that distinguish the PAM50 subtypes. Factors 1–3 clearly distinguished one or more subtypes, which motivated us to investigate what biological pathways each factor was associated with (Fig. 4 and Supplementary Fig. S4). Factor 1 was strongly associated with the Basal subtype and moderately associated with the HER2e subtype (Fig. 4A and B), while the negative factor loadings were enriched for Estrogen Response (ER) (Early and Late) pathways of the Molecular Signatures Database (MSigDB) Hallmark collection (Liberzon *et al.* 2015; Fig. 4Cand D).

The anti-correlation between the ER pathways and the Basal subtype, the highest scoring Factor 1 subtype, reflects that it is most strongly associated with a triple-negative profile for Estrogen, Progesterone, and the HER2e receptor (Prat *et al.* 2013). Whereas HER2e-classified samples were predominantly hormone receptor (HR) negative, a proportion is also HR positive (Bastien *et al.* 2012, Prat *et al.* 2015). LumA and LumB are hormone receptor positive (HR) and retain expression of ER, and the Progesterone receptor (PR) in the case of LumA, and in some proportion of LumB (Prat *et al.* 2015). Indeed, genes *XBP1*, *AGR2*, and *CA12* which yielded high posterior selection probabilities as well as the strongest negative projection coefficients in Factor 1 (Fig. 4E, Supplementary Table S1) for the Estrogen Response (Late) pathway were all associated with a breast cancer Steroid Responsiveness (SR) module that indicates functional steroid response (Fredlund *et al.* 2012). Genes *XBP1* and *MYB* were in the top 20% of the ER Early and Late pathway genes with respect to the SPEAR projection coefficient magnitude. *MYB* is a direct target of Estrogen signaling and is overexpressed in most ER+ cancers (Gonda *et al.* 2008), and both genes were also identified as part of a luminal expression signature (Cancer Genome Atlas Network 2012).

The positive association of Factor 1 with the MYC Targets V1 Hallmark pathway is consistent with the association of MYC signaling and Basal subtypes identified in earlier TCGA and other analyses (Xu *et al.* 2010). Additionally, four of the miRNAs associated with Factor 1, *hsa-mir-18a*, *hsa-mir-10a*, *hsa-mir135b*, *hsa-mir-577*, were identified in a miRNA analysis of TCGA data to have diagnostic significance for triple-negative breast cancer (Fan and Liu 2019). *Hsa-mir-18a* has been shown in independent datasets to downregulate ERα (Klinge 2012) and is associated with worse overall survival (Luengo-Gil *et al.* 2019). Hypomethylation of *MIA*, a PAM50 gene, which has the second highest in magnitude SPEAR projection coefficient for Factor 1, has been associated with the Basal subtype in TCGA and independent data (Bardowell *et al.* 2013).

Factor 1 distinguished Basal-like and HER2e subtypes from LumA/LumB, while Factors 2 and 3 distinguished LumA from the other subtypes. Pathways enriched in Factor 2 were associated with genes that are more highly expressed in LumA, whereas pathways in Factor 3 were associated with genes that are downregulated in LumA (Fig. 4A and B). The strongest signal in Factor 2 was in the Epithelial Mesenchymal Transition (EMT) Hallmark pathway, and in Factor 3 the G2M Checkpoint Hallmark pathway (Fig. 4C). It has been previously observed that LumB tumors are enriched in proliferation and cell-cycle associated genes in comparison to LumA (Prat *et al.* 2015), whereas the positive association of LumA with an EMT phenotype is somewhat surprising, as LumA tumors are generally considered to be associated with a stronger epithelial phenotype in comparison to the Basal-like subtype (Felipe Lima *et al.* 2016). The role of EMT has been primarily studied in non-luminal tumors^28^, and therefore the presence of this pathway distinguishing the subtypes warrants further investigation with respect to the underlying biology. Interestingly, the miRNAs with the top magnitude projection coefficients in Factor 2 and which have a positive association with the factor, *hsa-mir199a/b*, have been associated with EMT (Drago-García *et al.* 2017; Wang *et al.* 2019). Overall, the molecular signatures identified via SPEAR factorization of the TCGA-BC data provided both well-documented and novel associations with PAM50 breast cancer subtypes.

### 3.4 SPEAR identifies multi-omics factors and pathways associated with COVID-19 severity

**Figure 5.**
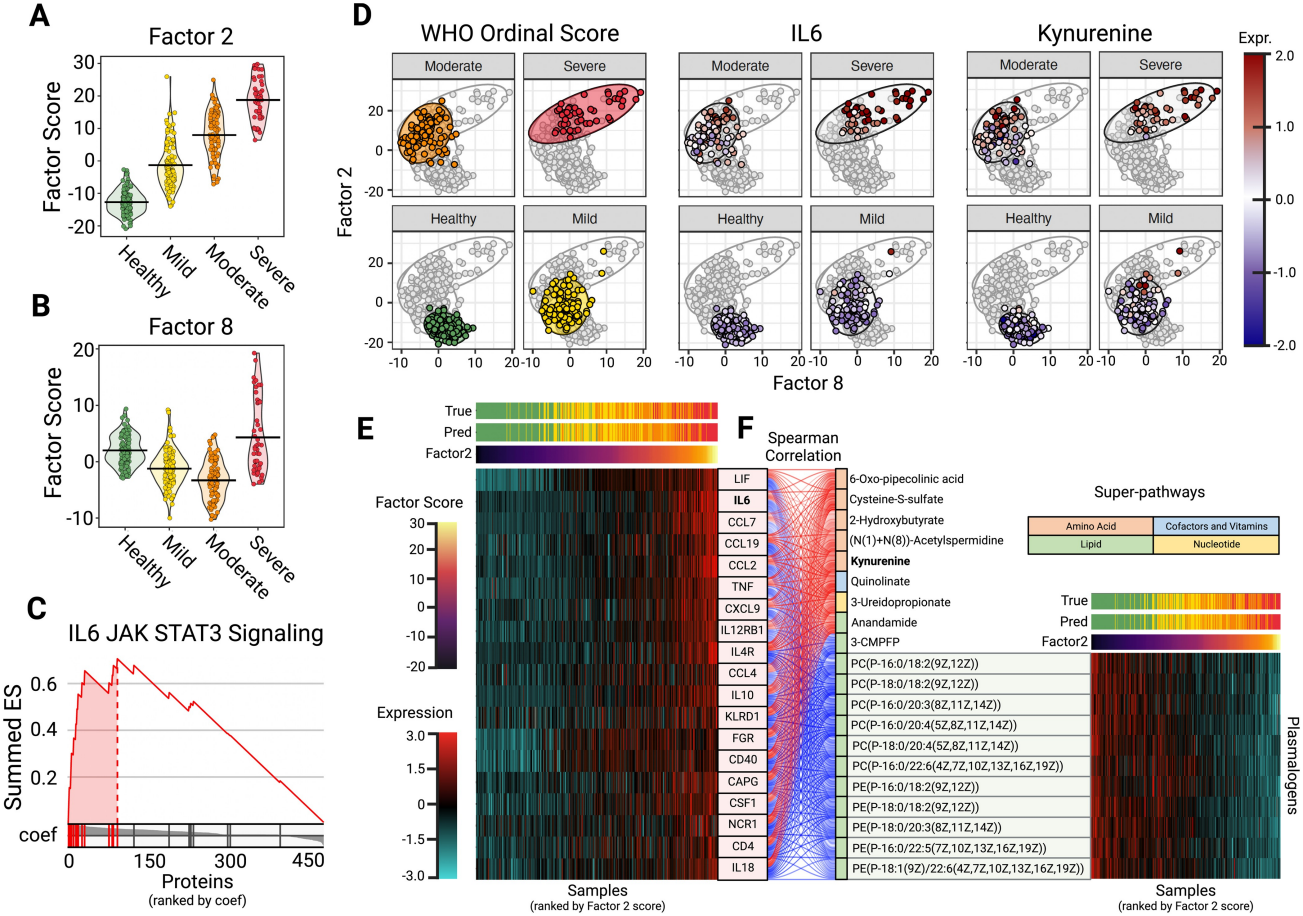
Downstream COVID-19 dataset analysis. (A) Grouped violin plot of factor 2 scores (*y*-axis) and simplified WHO score (*x*-axis), with group means marked with a line. (B) Grouped violin plot of factor 8 scores (y-axis) and simplified WHO score (*x*-axis), with group means marked with a line. (C) GSEA plot for IL6 JAK STAT3 Signaling Hallmark pathway for SPEAR Factor 2. Proteins are ranked by their assigned projection coefficient from SPEAR factor 2. (D) Embedding of samples by factor 8 (*x*-axis) and factor 2 (*y*-axis) scores. Samples are colored by WHO Ordinal Score, normalized IL6 expression, and normalized kynurenine expression. (E) Heatmap showing normalized expressions for proteins involved in the IL6 JAK STAT3 Signaling Hallmark pathway. Samples were ranked by Factor 2 score (*x*-axis) and proteins were ranked by projection coefficient (*y*-axis). Also shown are corresponding true patient severity scores (True) and SPEAR (sd) predicted severity scores (Pred). (F) Alluvial plot showing correlation between the IL6 JAK STAT3 proteins and the top metabolite features contributing to SPEAR Factor 2. Metabolites are grouped by positive/negative correlation and super-pathway. Also shown are normalized plasmalogen expressions of samples ranked by Factor 2 score (*x*-axis)

On the COVID-19 dataset, SPEAR identified several factors that were significantly associated with the WHO ordinal severity score (Fig. 5 and Supplementary Fig. S5). Investigation of the association of each factor with the COVID-19 severity revealed that the Factor 2 score showed a positive ordinal association (Fig. 5A). SPEAR also identified several factors that were heavily associated with identifying COVID-19 severities, including Factor 1 score for mild severity and Factor 8 for moderate severity (Fig. 5B and Supplementary Fig. S4). Embedding the samples by Factors 2 and 8 scores revealed a trajectory for SARS-CoV-2 severities (Fig. 5D). Calculation of the variance explained for these three factors revealed that Factor 2 had the largest influence from both assays (16% proteomics, 11% metabolomics) compared to Factor 1 (20% proteomics, 1% metabolomics) and Factor 8 (1% proteomics, 2% metabolomics) (Supplementary Fig. S5). We opted to further investigate Factor 2 due to its larger multi-omics influences.

Proteomic enrichment analysis of Factor 2 identified several significant pathways from the MSigDB Hallmark pathway database (Supplementary Table S2). One pathway enriched in Factor 2 was the Janus kinase Signal Transducer and Activator of Transcription (JAK-STAT) signaling pathway (Seif *et al.* 2017) (Fig. 5C). Pathway member Interleukin 6 (IL-6) was also found to be a key contributor to the Factor 2 score, with the second highest projection coefficient (Fig. 5E and Supplementary Table S3). IL-6 is a proinflammatory cytokine proposed as an inflammatory biomarker for COVID-19 severity (Azevedo *et al.* 2021) and is an activator of the JAK-STAT signaling pathway. The JAK-STAT signaling pathway is involved in immune regulation, lymphocyte growth and differentiation, and promotes oxidative stress, serving as an attractive therapeutic target for COVID-19 treatment (Luo *et al.* 2020).

The Factor 2 metabolic signature, extracted via automatic feature selection of the top contributing metabolites to the Factor 2 score, identified members of the amino acid, cofactors and vitamins, lipid, and nucleotide super-pathways (Fig. 5F). Kynurenine, a key contributor to the Factor 2 score, is a known marker of severe/fatal COVID-19 trajectory (Danlos *et al.* 2021, Mangge *et al.* 2021). The tryptophan/kynurenine (Trp/Kyn) pathway is activated by inflammatory cytokines (Almulla *et al.* 2022), which is consistent with its positive correlation with the cytokines of Factor 2 such as IL-6 (Fig. 5F).

Several plasmalogens, including phosphatidylcholines (PCs) and phosphatidylethanolamines (PEs) were also found to be inversely associated with the ordinal severity trend of Factor 2 (Fig. 5F). Plasmalogens are plasma-borne antioxidant phospholipids that provide endothelial protection during oxidative stress (Messias *et al.* 2018), which could account for the inverse association with the JAK-STAT signaling pathway proteins. Our multi-omics results further support the utility of plasmalogens as prognostic indicators of COVID-19 severity (Pike *et al.* 2022).

## 4 Discussion

SPEAR extracts interpretable multi-omics signatures from predictive factors via supervised dimensionality reduction and can model multiple types of responses, including Gaussian, multinomial, and ordinal. We have compared SPEAR to state-of-art linear dimension reduction methods as well as direct predictive models via Lasso regression in both simulated and real datasets. Unlike similar probabilistic factor models such as MOFA and iClusterBayes, the supervised SPEAR framework integrates the response of interest into the dimensionality reduction via the weight parameter w, resulting in factors containing signals from both the multi-omics assays and the response when supported by the data. While the optimal value of w can vary, the SPEAR algorithm provides an automatic choice of w that achieves good empirical prediction performance via cross-validation. In addition, SPEAR estimates analyte significance probabilities per factor, eliminating the need to perform feature selection via the loading coefficients entirely.

Defining the number of factors to be constructed can be critical when applying factor model-based methods. We have observed that underestimating the rank of a SPEAR model can lead to poor performance at higher values of w. To this end, SPEAR begins with an adaptive factor rank estimation that minimizes a debiased reconstruction error of X to estimate a sufficient number of factors required to accurately represent a multi-omics dataset (Supplementary Methods A2). While this approximation cannot guarantee true-rank estimation, it does return a satisfactory rank for dimensionality reduction via SPEAR from our experience. This approach is not restrictive to SPEAR and can be incorporated into other dimensionality reduction pipelines where the optimal number of factors to use is unknown.

While generalizable to many types of multi-omics, SPEAR does assume a linear dependence of the latent factors on the features. As such, the application of SPEAR to nonlinear analyte dependence may produce undesirable results. This could be mediated by appropriately preprocessing nonnormal multi-omics assays through transformations. A more data-adaptive solution would be to model the non-linear relationship between the features and factors via the generalized additive model (Hastie and Tibshirani 1995).

In conclusion, the SPEAR model decomposes high-dimensional multi-omics datasets into interpretable low-dimensional factors with high predictive power without the need for parameter tuning. SPEAR returns both sparse (regression) and full (projection) coefficients as well as feature-wise posterior probabilities used to assign analyte significance. SPEAR is currently hosted as a publicly available R-package at https://bitbucket.org/kleinstein/SPEAR

## Supplementary Material

btae202_Supplementary_Data

## Data Availability

No new data were generated or analysed in support of this research.

## References

[btae202-B1] Almulla AF , Supasitthumrong T, Tunvirachaisakul C et al The tryptophan catabolite or kynurenine pathway in COVID-19 and critical COVID-19: a systematic review and meta-analysis. BMC Infect Dis 2022;22:615. 35840908 10.1186/s12879-022-07582-1 PMC9284970

[btae202-B2] Argelaguet R , Arnol D, Bredikhin D et al MOFA+: a statistical framework for comprehensive integration of multi-modal single-cell data. Genome Biol 2020;21:111. 32393329 10.1186/s13059-020-02015-1 PMC7212577

[btae202-B3] Azevedo RB , Botelho BG, Hollanda JVGd et al Covid-19 and the cardiovascular system: a comprehensive review. J Hum Hypertens 2021;35:4–11. 32719447 10.1038/s41371-020-0387-4 PMC7384729

[btae202-B4] Banoth B , Cassel SL. Mitochondria in innate immune signaling. Transl Res 2018;202:52–68. 30165038 10.1016/j.trsl.2018.07.014 PMC6218307

[btae202-B5] Bardowell SA , Parker J, Fan C et al Differential methylation relative to breast cancer subtype and matched normal tissue reveals distinct patterns. Breast Cancer Res Treat 2013;142:365–80. 24212716 10.1007/s10549-013-2738-0 PMC3832780

[btae202-B6] Bastien RRL , Rodríguez-Lescure Á, Ebbert MTW et al PAM50 breast cancer subtyping by RT-qPCR and concordance with standard clinical molecular markers. BMC Med Genomics 2012;5:44. 23035882 10.1186/1755-8794-5-44 PMC3487945

[btae202-B7] Bhattacharya S , Andorf S, Gomes L et al ImmPort: disseminating data to the public for the future of immunology. Immunol Res 2014;58:234–9. 24791905 10.1007/s12026-014-8516-1

[btae202-B8] Bodkin N , Ross M, McClain MT et al Systematic comparison of published host gene expression signatures for bacterial/viral discrimination. Genome Med 2022;14:18. 35184750 10.1186/s13073-022-01025-x PMC8858657

[btae202-B9] Bolen CR , Ding S, Robek MD et al Dynamic expression profiling of type I and type III interferon-stimulated hepatocytes reveals a stable hierarchy of gene expression. Hepatology 2014;59:1262–72. 23929627 10.1002/hep.26657 PMC3938553

[btae202-B10] Cancer Genome Atlas Network Comprehensive molecular portraits of human breast tumours. Nature 2012;490:61–70. 23000897 10.1038/nature11412 PMC3465532

[btae202-B11] Cantini L , Zakeri P, Hernandez C et al Benchmarking joint multi-omics dimensionality reduction approaches for the study of cancer. Nat Commun 2021;12:124. 33402734 10.1038/s41467-020-20430-7 PMC7785750

[btae202-B12] Chawla DG , Cappuccio A, Tamminga A et al Benchmarking transcriptional host response signatures for infection diagnosis. Cell Syst 2022;13:974–88.e7. 36549274 10.1016/j.cels.2022.11.007 PMC9768893

[btae202-B13] Danlos F-X , Grajeda-Iglesias C, Durand S et al Metabolomic analyses of COVID-19 patients unravel stage-dependent and prognostic biomarkers. Cell Death Dis 2021;12:258–11. 33707411 10.1038/s41419-021-03540-y PMC7948172

[btae202-B14] DeLong ER , DeLong DM, Clarke-Pearson DL et al Comparing the areas under two or more correlated receiver operating characteristic curves: a nonparametric approach. Biometrics 1988;44:837–45. 3203132

[btae202-B15] Drago-García D , Espinal-Enríquez J, Hernández-Lemus E et al Network analysis of EMT and MET micro-RNA regulation in breast cancer. Sci Rep 2017;7:13534. 29051564 10.1038/s41598-017-13903-1 PMC5648819

[btae202-B16] Fan C , Liu N. Identification of dysregulated microRNAs associated with diagnosis and prognosis in triple-negative breast cancer: an in silico study. Oncol Rep 2019;41:3313–24. 30942465 10.3892/or.2019.7094

[btae202-B17] Felipe Lima J , Nofech-Mozes S, Bayani J et al EMT in breast Carcinoma-A review. J Clin Med 2016;5:E65. 10.3390/jcm5070065 PMC4961996 27429011

[btae202-B18] Fourati S , Tomalin LE, Mulè MP; Human Immunology Project Consortium (HIPC) et al Pan-vaccine analysis reveals innate immune endotypes predictive of antibody responses to vaccination. Nature Immunology 2022;23:1777–87. 36316476 10.1038/s41590-022-01329-5 PMC9747610

[btae202-B19] Fredlund E , Staaf J, Rantala JK et al The gene expression landscape of breast cancer is shaped by tumor protein p53 status and epithelial-mesenchymal transition. Breast Cancer Res 2012;14:R113. 22839103 10.1186/bcr3236 PMC3680939

[btae202-B21] Gonda TJ , Leo P, Ramsay RG et al Estrogen and MYB in breast cancer: potential for new therapies. Expert Opin Biol Ther 2008;8:713–7. 18476782 10.1517/14712598.8.6.713

[btae202-B23] Hagan T , Gerritsen B, Tomalin LE; Human Immunology Project Consortium (HIPC) et al Transcriptional atlas of the human immune response to 13 vaccines reveals a common predictor of vaccine-induced antibody responses. Nat Immunol 2022;23:1788–98. 36316475 10.1038/s41590-022-01328-6 PMC9869360

[btae202-B25] Hastie T , Tibshirani R. Generalized additive models for medical research. Stat Methods Med Res 1995;4:187–96. 8548102 10.1177/096228029500400302

[btae202-B26] Klinge CM. miRNAs and estrogen action. Trends Endocrinol Metab 2012;23:223–33. 22503553 10.1016/j.tem.2012.03.002 PMC3348384

[btae202-B27] Lazear HM , Schoggins JW, Diamond MS et al Shared and distinct functions of type I and type III interferons. Immunity 2019;50:907–23. 30995506 10.1016/j.immuni.2019.03.025 PMC6839410

[btae202-B28] Li W , Zhang S, Liu C-C et al Identifying multi-layer gene regulatory modules from multi-dimensional genomic data. Bioinformatics 2012;28:2458–66. 22863767 10.1093/bioinformatics/bts476 PMC3463121

[btae202-B29] Liberzon A , Birger C, Thorvaldsdóttir H et al The molecular signatures database (MSigDB) hallmark gene set collection. Cell Syst 2015;1:417–25. 26771021 10.1016/j.cels.2015.12.004 PMC4707969

[btae202-B30] Luengo-Gil G , García-Martínez E, Chaves-Benito A et al Clinical and biological impact of miR-18a expression in breast cancer after neoadjuvant chemotherapy. Cell Oncol (Dordr) 2019;42:627–44. 31115881 10.1007/s13402-019-00450-2 PMC12994329

[btae202-B31] Luo W , Li Y-X, Jiang L-J et al Targeting JAK-STAT signaling to control cytokine release syndrome in COVID-19. Trends Pharmacol Sci 2020;41:531–43. 32580895 10.1016/j.tips.2020.06.007 PMC7298494

[btae202-B32] Mangge H , Herrmann M, Meinitzer A et al Increased kynurenine indicates a fatal course of COVID-19. Antioxidants 2021;10:1960. 34943063 10.3390/antiox10121960 PMC8750518

[btae202-B33] Messias MCF , Mecatti GC, Priolli DG et al Plasmalogen lipids: functional mechanism and their involvement in gastrointestinal cancer. Lipids Health Dis 2018;17:41. 29514688 10.1186/s12944-018-0685-9 PMC5842581

[btae202-B34] Mo Q , Shen R, Guo C et al A fully Bayesian latent variable model for integrative clustering analysis of multi-type omics data. Biostatistics 2018;19:71–86. 28541380 10.1093/biostatistics/kxx017 PMC6455926

[btae202-B35] Nakaya HI , Wrammert J, Lee EK et al Systems biology of vaccination for seasonal influenza in humans. Nat Immunol 2011;12:786–95. 21743478 10.1038/ni.2067 PMC3140559

[btae202-B36] Parker JS , Mullins M, Cheang MCU et al Supervised risk predictor of breast cancer based on intrinsic subtypes. J Clin Oncol 2009;27:1160–7. 19204204 10.1200/JCO.2008.18.1370 PMC2667820

[btae202-B37] Pike DP , McGuffee RM, Geerling E et al Plasmalogen loss in sepsis and SARS-CoV-2 infection. Front Cell Dev Biol 2022;10:912880. 35784479 10.3389/fcell.2022.912880 PMC9242022

[btae202-B38] Prat A , Pineda E, Adamo B et al Clinical implications of the intrinsic molecular subtypes of breast cancer. Breast 2015;24:S26–35. 26253814 10.1016/j.breast.2015.07.008

[btae202-B39] Prat A , Adamo B, Cheang MCU et al Molecular characterization of basal-like and non-basal-like triple-negative breast cancer. Oncologist 2013;18:123–33. 23404817 10.1634/theoncologist.2012-0397 PMC3579595

[btae202-B40] Ramilo O , Allman W, Chung W et al Gene expression patterns in blood leukocytes discriminate patients with acute infections. Blood 2007;109:2066–77. 17105821 10.1182/blood-2006-02-002477 PMC1801073

[btae202-B41] Rubio-Rivas M , Mora-Luján JM, Formiga F, SEMI-COVID-19 Network et al WHO ordinal scale and inflammation risk categories in COVID-19. comparative study of the severity scales. J Gen Intern Med 2022;37:1980–7. 35396659 10.1007/s11606-022-07511-7 PMC8992782

[btae202-B42] Seif F , Khoshmirsafa M, Aazami H et al The role of JAK-STAT signaling pathway and its regulators in the fate of T helper cells. Cell Commun Signal 2017;15:23. 28637459 10.1186/s12964-017-0177-y PMC5480189

[btae202-B43] Singh A , Shannon CP, Gautier B et al DIABLO: an integrative approach for identifying key molecular drivers from multi-omics assays. Bioinformatics 2019;35:3055–62. 30657866 10.1093/bioinformatics/bty1054 PMC6735831

[btae202-B45] Su Y , Chen D, Yuan D, ISB-Swedish COVID19 Biobanking Unit et al Multi-omics resolves a sharp disease-state shift between mild and moderate COVID-19. Cell 2020;183:1479–95.e20. 33171100 10.1016/j.cell.2020.10.037 PMC7598382

[btae202-B46] Tenenhaus A , Philippe C, Guillemot V et al Variable selection for generalized canonical correlation analysis. Biostatistics 2014;15:569–83. 24550197 10.1093/biostatistics/kxu001

[btae202-B47] Tenenhaus A , Tenenhaus M. Regularized generalized canonical correlation analysis. Psychometrika 2011;76:257–84. 10.1007/s11336-017-9573-x 28536930

[btae202-B49] Tibshirani R. Regression shrinkage and selection via the lasso. Journal of the Royal Statistical Society: Series B (Methodological) 1996;58:267–88.

[btae202-B50] Walker KA , Chen J, Shi L et al Proteomics analysis of plasma from middle-aged adults identifies protein markers of dementia risk in later life. Sci Transl Med 2023;15:eadf5681. 37467317 10.1126/scitranslmed.adf5681 PMC10665113

[btae202-B52] Wang Q , Ye B, Wang P et al Overview of microRNA-199a regulation in cancer. Cancer Manag Res 2019;11:10327–35. 31849522 10.2147/CMAR.S231971 PMC6911337

[btae202-B54] Xu J , Chen Y, Olopade OI et al MYC and breast cancer. Genes Cancer 2010;1:629–40. 21779462 10.1177/1947601910378691 PMC3092228

[btae202-B55] Yu F , Quan F, Xu J et al Breast cancer prognosis signature: linking risk stratification to disease subtypes. Brief Bioinform 2019;20:2130–40. 30184043 10.1093/bib/bby073

