# Supplementary material for: A supervised Bayesian factor model for the identification of multi-omics signatures
Source: Bioinformatics. 2024 Apr 11;40(5):btae202. doi: 10.1093/bioinformatics/btae202 (PMC11078774; doi:10.1093/bioinformatics/btae202)
Supplement: btae202_Supplementary_Data [file btae202_supplementary_data.pdf]

In this supplementary material, we cover (1) full SPEAR model details, (2) algorithm details for variational Bayesian model, and (3) additional empirical results.

For analysis code and supplementary tables, please access the SPEAR Supplementary Code Repository:

(https://bitbucket.org/kleinstein/projects/src/master/Gygi2024_SPEAR/)

Contents

A SPEAR overview **2**
- A.1 Modeling of various response types . . . 3
- A.2 Rank selection and weight tuning . . . 4

B Parameter estimation for Gaussian response **6**
- B.1 Review of the mean-field approximation . . . 6
- B.2 Variational distributions for SPEAR . . . 7
- B.3 Formula for different expectations . . . 7
- B.4 Parameter update . . . 8

C Parameter estimation with non-Gaussian response **12**
- C.1 Lower Bounds of ELBO . . . 13
- C.2 Two-class logistic regression . . . 13
- C.3 Ordinal logistic regression . . . 14
- C.4 Multi-class logistic regression . . . 15

D Additional results on synthetic data **16**
- D.1 Gaussian Simulation . . . 16
- D.2 Ordinal Simulation . . . 19
- D.3 Multinomial Simulation . . . 20

E Additional results on real data **21**
- E.1 TCGA-BC dataset background and preprocessing . . . 21
- E.2 COVID-19 dataset background and preprocessing . . . 22
- E.3 SPEAR Factor Influence on Prediction . . . 22

A. SPEAR overview

Fixing the model rank K and the weight parameter w, SPEAR considers the following weighted likelihood for the data given the latent factor $\mathbf{U}$ and the model parameters:

$$P(\mathbf{X}, \mathbf{Y}|\mathbf{U}, \Theta) = \left(\prod_{i=1}^{N}\prod_{j=1}^{p} P^w(x_{i,j}|\mathbf{u}_i, \Theta)\right) \times \left(\prod_{i=1}^{N}\prod_{j=1}^{\overline{p}} P(y_{i,j}|\mathbf{u}_i, \Theta)\right). \tag{1}$$

Let $\mathbf{X}$ and $\mathbf{Y}$ represent the high-dimensional multi-omics assays and low-dimensional response(s) respectively and let $\mathbf{Z}$ represent either $\mathbf{X}$ if completely observed or alternatively the fully imputed version of $\mathbf{X}$ if there are missing data. We use $P(.)$ to denote a likelihood function for either a continuous or discrete variable. For the latent factor $\mathbf{U}$, we model it as a noisy realization of a linear function of $\mathbf{Z}$, and let

$$\mathbf{U} = \mathbf{Z}\boldsymbol{\beta} + \boldsymbol{\mathcal{E}}, \tag{2}$$

where $\boldsymbol{\mathcal{E}}_{ik} \sim \mathcal{N}(0, 1)$. We introduce $\boldsymbol{\mathcal{E}}$ here for stability that can happen when $Z\boldsymbol{\beta}$ becomes very small. $\Theta = \left\{\sigma^2, \boldsymbol{\beta}, \mathbf{B}, \overline{\mathbf{B}}\right\}$ is the collection of all model parameters, with β representing the regression coefficients as well as $\mathbf{B}$ and $\overline{\mathbf{B}}$ representing the projection coefficients of $\mathbf{X}$ and $\mathbf{Y}$ respectively. . Let $\Gamma(.|a, c)$ and $\text{Beta}(.|a, c)$ represent the Gamma and Beta distributions with parameters (a, c):

$$\begin{aligned}\Gamma(x|a, c) =& \frac{c^\alpha}{\Gamma(\alpha)} x^{\alpha-1} \exp(-cx)\\ \text{Beta}(x|a, c) =& \frac{\Gamma(a+c)x^{a-1}(1-x)^{c-1}}{\Gamma(a)\Gamma(c)}\end{aligned}$$

Let $p(.)$ be a density function; for continuous variables, we use the following priors for the model parameters:

- Inverse Gamma prior for variances. Let σ_j^2 be the variance of X_j. For convenience, we let $\nu_j = \frac{1}{\sigma_j^2}$ and will use ν_j from here.

$$p(\nu_j) = \Gamma(\nu_j|a_0, c_0) \tag{3}$$

- By default, we set features from the same assay as one group. For $j = 1, \ldots, p$, let $\beta_{jk} = \hat{\beta}_{jk}\gamma_{jk}$, where $\hat{\beta}_{jk} \sim \mathcal{N}(0, \frac{1}{\tau_{g_jk}})$ and $\gamma_{jk} \sim Binary(\pi_{g_jk})$. We model different groups to have different prior non-zero probabilities and variances when being non-zero. Consider the case where features can be grouped into G different groups, and g_j is the group identifier for feature j. Symmetrically, we let $B_{jk} = \hat{B}_{jk}s_{jk}$ where $\hat{B}_{jk} \sim \mathcal{N}(0, \frac{1}{\tau_{g_jk}})$ and $s_{jk} \sim Binary(\pi_{g_jk})$ for $j = 1, \ldots, p$.

- We do not impose sparsity for $\overline{B}_{jk}$, $j > p$, which corresponds to projection coefficients of Y_j onto the factors, and model $\overline{B}_{jk} \sim \mathcal{N}(0, \frac{1}{\tau_0})$. Since Y is low-dimensional, we aim to model all of them.

To avoid the need of extensive parameter tuning, we further model the priors τ, π with some hyper-prior distributions for group $g = 1, \ldots, G$ and factor $k = 1, \ldots, K$:

- We model τ_{gk} with a Gamma prior,

$$P(\tau_{gk}) = \Gamma(\tau_{gk}|a_1, c_1).$$

- We model π_{gk} with a Beta prior,

$$P(\pi_{gk}) = \text{Beta}(\pi_{gk}|a_2, c_2).$$

The hyper prior modeling allows τ_{gk} and π_{gk} to be tuning free. As a result, such a model is referred to an ARD (automatic relevance determining) model since feature sparsity and effect size for each group is estimated from the data (David Wipf [2007]).

In this paper, we model the features X as Gaussian but allow Y to be either Gaussian, categorical or ordinal, which are common data types in practice. We can also model X to be different data types, although in practice X is usually continuous or binary. For both cases, modeling X as Gaussian is reasonable for signal extraction purpose. We allow for more flexibility in modeling Y for the purpose of both better signal extraction and interpretability. When Y is a multiclass categorical response or an ordinal response, modeling Y as Gaussian may lead to worse estimation (if Y is highly non-linear in its nominal values in the ordinal case, or having co-linearity in the multinomial case). The resulting prediction can also be more difficult to interpret.

A.1. Modeling of various response types

In Eq. (1), $\overline{p}$ is the dimension of response so that we can deal with multi-dimensional response y as well. For convenience, we discuss only the one-dimensional response setting with $\overline{p} = 1$ and denote y_{i1} by y_i. Modeling of the multi-dimensional response is a simple extension of the one-dimensional problem. By taking proper forms for $P(y_i|\mathbf{u}, \Theta)$, we can flexibly model different response types. SPEAR support four response types:

Continuous response: We consider a Gaussian model for continuous response y:

$$P(y_i|\mathbf{u}_i, \Theta) = \frac{1}{\sqrt{2\pi\bar{\sigma}^2}} \exp(-\frac{(y_i - \mathbf{u}_i^\top \bar{B})^2}{2\bar{\sigma}^2})$$

where $\bar{B}$ is the projection coefficient from y onto $\mathbf{U}$, u_i is the ith row vector of $\mathbf{U}$, and $\bar{\sigma}^2$ is the variance of noise in y.

Two-class categorical response We model the two-class categorical response $y_i \in \{0, 1\}$ using the logistic regression:

$$P(y_i|\mathbf{u}_i, \Theta) = \left[\frac{1}{1 + \exp(-\mathbf{u}_i^\top \bar{B} - \alpha)}\right]^{y_i} \left[\frac{1}{1 + \exp(\mathbf{u}_i^\top \bar{B} + \alpha)}\right]^{1-y_i},$$

where α is the intercept in the logistic regression.

Ordinal response: We consider an ordinal logistic model for modeling the ordinal response. Suppose that there are M ordered classes and $y_i \in \{1, \ldots, M\}$, then

$$P(y_i|\mathbf{u}_i, \Theta) = \prod_{m=1}^{M} [\mathbf{P}(y_i \geq m|\mathbf{u}_i, \Theta) - \mathbf{P}(y_i \geq m + 1|\mathbf{u}_i, \Theta)]^{\mathbb{1}_{y_i = m}}$$

$$= \prod_{m=1}^{M} \left[\frac{1}{1+\exp(\mathbf{u}_i^\top \bar{B} + \alpha_m)} - \frac{1}{1+\exp(\mathbf{u}_i^\top \bar{B} + \alpha_{m+1})} \right]^{\mathbb{1} y_i = m},$$

where $\infty = \alpha_1 > \ldots > \alpha_M > \alpha_{M+1} = -\infty$ are the "cuts" along the direction $\mathbf{u}_i^\top \bar{B}$ that determines the probability of falling into each class, and $\mathbb{1}\{y_i = m\}$ is the indicator function for $y_i = m$.

Multi-class categorical response: We model the multi-class categorical response $y_i \in \{1, \ldots, M\}$ using the multinomial logistic regression. In this case $\bar{B} \in \mathbb{R}^{K \times M}$ is a matrix with m^{th} column representing the coefficient for class m:

$$P(y_i|\mathbf{u}_i, \Theta) = \prod_{m=1}^{M} \left[\frac{\exp(\mathbf{u}_i^\top \bar{B}_m + \alpha_m)}{\sum_{m'=1}^{M} \exp(\mathbf{u}_i^\top \bar{B}_{m'} + \alpha_{m'})} \right]^{\mathbb{1}\{y_i = m\}}$$

In Appendix B, we first derive the iterative parameter estimation procedures for the Gaussian data, and we then move to the non-Gaussian case in Appendix C.

A.2. Rank selection and weight tuning

In A.1, we described the SPEAR model with fixed weight parameter w and rank K. Here, we give more details on our default choice of w and K.

Tuning the weight parameter w: We tune the weight parameter adaptively from the data based on cross-validation on the response's deviance loss. To reduce the computational burden, we adopt the warm-start strategy: when running the model with a lower weight, we use model parameters from the higher weight before it as the starting point. Suppose that we divide the data into T random folds $\cup_{t=1}^{T} \mathcal{D}_t$. Let $\hat{\Theta}(w;t)$ be the estimated posterior means for model parameters using data excluding fold t. Let $D(y_i, x_i|\hat{\Theta}(w;t))$ be the deviance loss evaluation at sample i using the parameter $\hat{\Theta}(w;t)$, e.g, for Gaussian response, we have

$$D(y_i, x_i|\hat{\Theta}(w,t)) = \|y_i - [x_i^\top \hat{\boldsymbol{\beta}}(w,t)]\hat{\bar{\mathbf{B}}}(w,t)\|_2^2$$

where $\hat{\boldsymbol{\beta}}(w,t)$ and $\hat{\bar{\mathbf{B}}}(w,t)$ are the estimated posterior means for $\boldsymbol{\beta}$ and $\mathbf{B}$ using data excluding fold t and at weight parameter w. Then, we can choose w minimizing the average deviance loss:

$$D(Y, \mathbf{X}|\hat{\Theta}(w)) = \frac{1}{N} \sum_{t=1}^{T} \sum_{i \in \mathcal{D}_t} D(y_i, x_i|\hat{\Theta}(w,t)).$$

Let w^* be the weight that minimizes the cross-validation error. In the 1 standard deviation rule, we choose the largest weight w such that

$$D(Y, \mathbf{X}|\hat{\Theta}(w)) \leq D(Y, \mathbf{X}|\hat{\Theta}(w^*)) + \widehat{sd}\left(D(Y, \mathbf{X}|\hat{\Theta}(w^*)) \right),$$

where $\widehat{sd}\left(D(Y, \mathbf{X}|\hat{\Theta}(w^*)) \right)$ is the estiamted standard deviation of $D(Y, \mathbf{X}|\hat{\Theta}(w^*;t))$ by comparing the average deviance loss from different folds. We suggesting using w^* when the goal is for better prediction and using the 1sd rule when more structure in $\mathbf{X}$ is preferred.

Rank selection: SPEAR chooses a weight parameter adaptively after fixing the rank K. When there is strong factor structure in $\mathbf{X}$ that is irrelevant to Y, choosing a rank K too small can push our model towards small w, which can be unwanted in practice sometimes for interpretation. Here, we use recently developed statistical techniques and propose a simple novel approach as the default rank selection rule for SPEAR. We have assumed the following latent factor model for the features $\mathbf{X} \in \mathbb{R}^{N\times p}$:

$$\mathbf{X} = \mathbf{UB} + \mathbf{E},$$

where $\mathbf{E} \in \mathbb{R}^{N\times p}$ is the unstructured noise matrix and $E_{ij} \overset{i.i.d}{\sim} N(0, \sigma_j^2)$. Without loss of generality, we always standardize $\mathbf{X}$ to have mean 0 and variance 1.

Now, let's regenerate a new noise matrix $\tilde{\mathbf{E}}$ where $\tilde{E}_{ij} \overset{i.i.d}{\sim} N(0, \sigma_j^2)$ follows the same distribution of E_{ij}. A key observation is that

$$\mathbf{X}^{\kappa} = \mathbf{UB} + \mathbf{E} + \kappa\tilde{\mathbf{E}}$$

and

$$\mathbf{X}^{\frac{1}{\kappa}} = \mathbf{UB} + \mathbf{E} - \frac{1}{\kappa}\tilde{\mathbf{E}}$$

are marginally independent if we consider both the randomness in $\mathbf{E}$ and the randomness in $\tilde{\mathbf{E}}$. This is a direct result of the Gaussianity and the fact that the covariance between $\mathbf{X}^{\kappa}$ and $\mathbf{X}^{\frac{1}{\kappa}}$:

$$cov(\mathbf{X}^{\kappa}, \mathbf{X}^{\frac{1}{\kappa}}) = \mathbf{0}.$$

Following this observation, we propose the following rank selection approach: This idea

for $\ell = 1, \ldots, L$ **do**

Generate $i.i.d$ Gaussian noise $\tilde{\mathbf{E}}$, and form $\mathbf{X}^{\kappa}$, $\mathbf{X}^{\frac{1}{\kappa}}$ with $\kappa = 0.1$.

Perform PCA on $\mathbf{X}^{\kappa}$, and let $\hat{\mathbf{X}}[k]$ be the reconstructed feature matrix with rank k for $k = 1, \ldots, K_{\max}$.

Evaluate the reconstruction loss comparing $\hat{\mathbf{X}}[k]$ and $\mathbf{X}^{\frac{1}{\kappa}}$:

$$r_{kl} = \frac{1}{Np}\|\mathbf{X}^{\frac{1}{\kappa}} - \hat{\mathbf{X}}[k]\|_F^2$$

Select the rank with smallest average reconstruction loss

$$r_k = \frac{1}{L}\sum_{l=1}^{L} r_{kl}, \quad k^* = \arg\min_{k\in\{1,\ldots,K_{\max}\}} r_k$$

end

Algorithm 1: Rank selection in SPEAR

has been applied in the supervised regression problem for model selection (Guan and Tibshirani, 2020; Tian, 2020), but not to the rank selection.

In practice, we do not know the true noise level σ_j^2. Since we are most interested finding the strong factors, we can afford using a larger variance estimate. When replacing

σ_j^2 by $\hat{\sigma}_j^2$ with $\hat{\sigma}_j^2 > \sigma_j^2$, this will introduce negative correlation between $\mathbf{X}^{\kappa}$ and $\mathbf{X}^{\frac{1}{\kappa}}$, which in turn favors smaller K. However, when the factor is strong, we expect them to be found even in this conservative setting. As a result, in the default SPEAR algorithm, instead of trying to estimate σ_j^2 accurately, we use an aggressive estimate of $\hat{\sigma}_j^2 \in [0, 1]$, e.g., $\hat{\sigma}_j^2 = 0.9$ is our default value. In our simulation and empirical studies, we have selected the model rank using this default strategy. We observe it works well for both cases: it often selects the true ranks in simulations and it also selects ranks that do not push w to 0 in real data experiments.

B. Parameter estimation for Gaussian response

B.1. Review of the mean-field approximation

Let $\Theta = \{\theta_1, \ldots, \theta_T\}$ be the collection of T-block of parameters, and $\mathcal{D}$ represent the data. Our goal is to approximate the posterior distribution of Θ with a simpler function q and find q minimizing their KL divergence between these two distributions:

$$KL(q(\Theta)\|p(\Theta|\mathcal{D})) = \int q(\Theta) \log \frac{q(\Theta)}{p(\Theta|\mathcal{D})} d\Theta$$

Define the evidence lower bound (ELBO) to be the integral of the log ratio between the marginal density $p(\Theta, \mathcal{D})$ and $q(\Theta)$:

$$ELBO(q) = \int q(\Theta) \log \frac{p(\Theta, \mathcal{D})}{q(\Theta)} d\Theta = -KL(q(\Theta)\|p(\Theta|\mathcal{D})) + \log p(\mathcal{D}) \tag{4}$$

The log marginal likelihood on the data $\log p(\mathcal{D})$ is called the evidence. It measures how well our model describes the data and is a constant. Consequently, minimizing the KL-divergence between the variational distribution is equivalent of maximizing the ELBO. By choosing a proper variational distribution q such that the integral of the log joint density and $\log q$ over q is easy to calculate, we can find an optimal or local optimal solution to this maximization problem efficiently:

$$ELBO(q) = \int q(\Theta) \log p(\Theta, \mathcal{D}) - \int q(\Theta) \log q(\Theta)$$

The space of q often does not contain the actual posterior distribution $p(\Theta|\mathcal{D})$. However, variational Bayes has proven successful in many applied problems and researchers sometimes are willing to sacrifice accuracy for efficiency, especially in large-scale datasets.

One popular variational Bayesian method is mean field approximation, which fully decouples the parameters into several independent blocks and considers:

$$q(\Theta) = \prod_{j=1}^{p} q_{\alpha_j}(\theta_j)$$

where α_j is the parameter determining the distribution $q_{\alpha_j}(\theta_j)$. We can often iteratively update $q_{\alpha_j}(\theta_j)$ in a computational efficient manner given the distributions of other parameters:

$$\int q(\Theta) \left(\log p(\Theta, \mathcal{D}) - \log q(\Theta)\right)$$

$$\propto \int_j q_{\alpha_j}(\theta_j) \left(\left(\int_{\Theta_{-j}} q_{-j}(\Theta_{-j}) \log p(\Theta, \mathcal{D}) d\Theta_{-j} \right) - \log q_{\alpha_j}(\theta_j) \right) d\theta_j$$

Fixing others, we choose the parameters α_j that improve most the ELBO:

$$L(q) = \int q(\Theta) \log \frac{p(Y,\Theta)}{q(\Theta)} = E_q(\log p(Y|\Theta)) + \sum_{j=1}^{p} \left(E_{q_j} \log p(\theta_j) - E_{q_j} \log q(\theta_j) \right) \quad (5)$$

We can update the variational parameters $\alpha_1, \ldots, \alpha_p$ iteratively until the ELBO updates are deemed insignificant.

B.2. Variational distributions for SPEAR

Let $q(\Theta \cup \boldsymbol{\mathcal{E}})$ be the mean field approximation to the posterior distribution of $\Theta \cup \boldsymbol{\mathcal{E}}$. We assume q to be the product of a series of independent components:

$$q(\Theta \cup \boldsymbol{\mathcal{E}}) = \left(\sum_{k=1}^{K} \prod_{j=1}^{p} q(\beta_{jk}) \right) \times \left(\sum_{k=1}^{K} \prod_{j=1}^{p} q(B_{jk}) \right) \times \left(\prod_{j=1}^{\overline{p}} q(\bar{B}_j) \right) \times \left(\prod_{i=1}^{n} \prod_{k=1}^{K} q(\boldsymbol{\mathcal{E}}_{ik}) \right)$$
$$\times \left(\prod_{j=1}^{p} q(\nu_j) \right) \times \left(\prod_{j=1}^{\overline{p}} q(\bar{\nu}_j) \right) \times \left(\prod_{g=1}^{G} \prod_{k=1}^{K} q(\tau_{gk}) \right) \times \left(\prod_{g=1}^{G} \prod_{k=1}^{K} q(\pi_{gk}) \right)$$

The specific forms for each component are given below.

$$q(\bar{B}_{jk}) = \mathcal{N}(\bar{B}_{jk}|\bar{\mu}_{jk}, \bar{\zeta}_{jk}^2),\ q(\boldsymbol{\mathcal{E}}_{ik}) = \mathcal{N}(\boldsymbol{\mathcal{E}}_{ik}|0, e_{ik}),$$
$$q(\nu_j) = \Gamma(\nu_j|\tilde{a}_{j0}^X, \tilde{c}_{j0}^X),\ q(\bar{\nu}_j) = \Gamma(\nu_j|\tilde{a}_{j0}^Y, \tilde{c}_{j0}^Y),$$
$$q(\tau_{gk}) = \Gamma(\tau_{gk}|\tilde{a}_{gk1}, \tilde{c}_{gk1}),\ q(\pi_{gk}) = \Gamma(\pi_{gk}|\tilde{a}_{gk2}, \tilde{c}_{gk2}),$$

and also,

$$q(\beta_{jk}) \coloneqq q(\hat{\beta}_{jk}, \gamma_{jk}) = \begin{cases} \mathcal{N}(\hat{\beta}_{jk}|\mu_{jk}, \zeta_{jk}^2) & \text{if} \quad \gamma_{jk} = 1, \\ \mathcal{N}(\hat{\beta}_{jk}|0, \zeta_{0jk}^2) & \text{otherwise.} \end{cases}, \gamma_{jk} = \begin{cases} 1 & \text{with probability} \quad \omega_{jk}, \\ 0 & \text{with probability} \quad (1-\omega_{jk}) \end{cases}$$
$$q(B_{jk}) \coloneqq q(\hat{B}_{jk}, s_{jk}) = \begin{cases} \mathcal{N}(\hat{B}_{jk}|\tilde{\mu}_{jk}, \tilde{\zeta}_{jk}^2) & \text{if} \quad s_{jk} = 1, \\ \mathcal{N}(\hat{B}_{jk}|0, \tilde{\zeta}_{0jk}^2) & \text{otherwise.} \end{cases}, s_{jk} = \begin{cases} 1 & \text{with probability} \quad \tilde{\omega}_{jk}, \\ 0 & \text{with probability} \quad (1-\tilde{\omega}_{jk}) \end{cases}$$

B.3. Formula for different expectations

We use $\langle . \rangle$ to represent the expectation under the variational distribution. Then,

- $\langle \beta_{jk} \rangle = \langle \gamma_{jk} \rangle \langle \hat{\beta}_{jk} \rangle = \omega_{jk}\mu_{jk}$, $\langle \beta_{jk}^2 \rangle = \omega_{jk} \left(\mu_{jk}^2 + \zeta_{jk}^2 \right)$, $\langle \hat{\beta}_{jk}^2 \rangle = \langle \beta_{jk}^2 \rangle + (1-\omega_{jk})\zeta_{0jk}^2$.
- $\langle B_{jk} \rangle = \langle s_{jk} \rangle \langle \hat{B}_{jk} \rangle = \tilde{\omega}_{jk}\tilde{\mu}_{jk}$, $\langle B_{jk}^2 \rangle = \tilde{\omega}_{jk} \left(\tilde{\mu}_{jk}^2 + \tilde{\zeta}_{jk}^2 \right)$, $\langle \hat{B}_{jk}^2 \rangle = \langle B_{jk}^2 \rangle + (1-\tilde{\omega}_{jk})\tilde{\zeta}_{0jk}^2$.
- $\langle \boldsymbol{\mathcal{E}}_{ik} \rangle = 0$, $\langle \boldsymbol{\mathcal{E}}_{ik}^2 \rangle = e_{ik}$.

- $\langle u_{ik}\rangle = z_i^{\perp}\langle \boldsymbol{\beta}_k\rangle$, $\langle u_{ik}^2\rangle = (z_i^{\perp}\langle \boldsymbol{\beta}_k\rangle)^2 + \sum_{j=1}^{p} z_{ij}^2(\langle \boldsymbol{\beta}_k^2\rangle - \langle \boldsymbol{\beta}_k\rangle^2) + \langle \boldsymbol{\mathcal{E}}_{ik}^2\rangle$.

  We collect some results about the Gamma and Beta distributions below. Let x be a variable with density $\Gamma(x|a,c)$ or $\text{Beta}(x|a,c)$ depending on the context. Let $\Psi(a,c) = \frac{d\log\Gamma(a,c)}{da}$ be the first-order polyGamma distribution, then,

$$\mathbb{E}_{\Gamma}[x] = \frac{a}{c},\ \mathbb{E}_{\Gamma}[\log x] = \log c + \Psi(a,c),$$

$$\mathbb{E}_{\text{Beta}}[x] = \frac{a}{a+c},\ \mathbb{E}_{\text{Beta}}[\log x] = \Psi(a+c,1) - \Psi(a,1),\ \mathbb{E}_{\text{Beta}}[\log(1-x)] = \Psi(a+c,1) - \Psi(c,1).$$

  We can get the expectations $\langle \nu_j\rangle$, $\langle \bar{\nu}_j\rangle$, $\langle \tau_{gk}\rangle$, $\langle \log\tau_{gk}\rangle$, $\langle \pi_{gk}\rangle$, $\langle \log\pi_{gk}\rangle$, $\langle \log(1-\pi_{gk})\rangle$ straightforwardly based on the above formulas.

B.4. Parameter update

Update $Q(\beta_{jk})$. For any parameter θ, we use $L_0(\theta), L_1(\theta), L_2(\theta)$ to denote the logarithms of the prior, data likelihood and variational distribution throughout this supplement. For example, with respect to β_{jk}, we let $L_0(\beta_{jk})$, $L_1(\beta_{jk})$, and $L_2(\beta_{jk})$ denote its expected log prior, log data likelihood and log variational distribution respectively. Then,

$$L_0(\beta_{jk}) \propto -\frac{1}{2}\langle \hat{\beta}_{jk}^2\rangle\langle \tau_{g_jk}\rangle + \langle \gamma_{jk}\rangle\langle \ln\pi_{g_j\pi}\rangle + \langle \gamma_{jk}\rangle\langle \ln(1-\pi_{g_j\pi})\rangle, \tag{6}$$

$$L_1(\beta_{jk}) \propto -\frac{1}{2}\sum_{j'=1}^{p} w_{j'}\langle \nu_{j'}\rangle\langle \|\mathbf{X}_{j'} - \mathbf{U}\mathbf{B}_{j'}\|_2^2\rangle - \frac{1}{2}\sum_{j'=1}^{\bar{p}}\langle \bar{\nu}_{j'}\rangle\langle \|\mathbf{Y}_{j'} - \mathbf{U}\bar{\mathbf{B}}_{j'}\|_2^2\rangle, \tag{7}$$

$$L_2(\beta_{jk}) \propto -\omega_{jk}\frac{1+\ln\zeta_{jk}^2}{2} - (1-\omega_{jk})\frac{1+\ln\zeta_{0jk}^2}{2} + (1-\omega_{jk})\ln(1-\omega_{jk}) + \omega_{jk}\ln\omega_{jk}. \tag{8}$$

Re-arrange the terms for $L_1(\beta_{jk})$, we have

$$L_1(\beta_{jk}) \propto \sum_{i=1}^{n}(I_{i1} - I_{i2})\, z_{ij}\langle \beta_{jk}\rangle - \frac{1}{2}\sum_{i=1}^{n} I_{i3} z_{ij}^2\langle \beta_{jk}^2\rangle \tag{9}$$

where

$$I_{i1} = \sum_{j'=1}^{p}\langle \nu_{j'}\rangle w_{j'}\left\langle x_{ij'} - \sum_{k'\neq k} u_{ik'}B_{j'k'}\right\rangle\langle B_{j'k}\rangle + \sum_{j'=1}^{\bar{p}}\langle \bar{\nu}_{j'}\rangle\left\langle y_{ij'} - \sum_{k'\neq k} u_{ik'}\bar{B}_{j'k'}\right\rangle\left\langle \bar{B}_{j'k}\right\rangle$$

$$I_{i2} = \sum_{j'=1}^{p}\langle \nu_{j'}\rangle w_{j'}\left\langle u_{ik}^{\backslash j}\right\rangle\left\langle B_{j'k}^2\right\rangle + \sum_{j'=1}^{\bar{p}}\langle \bar{\nu}_{j'}\rangle\left\langle u_{ik}^{\backslash j}\right\rangle\left\langle \bar{B}_{j'k}^2\right\rangle,$$

$$I_{i3} = \sum_{j'=1}^{p}\langle \nu_{j'}\rangle w_{j'}\left\langle B_{j'k}^2\right\rangle + \sum_{j'=1}^{\bar{p}}\langle \bar{\nu}_{j'}\rangle\left\langle \bar{B}_{j'k}^2\right\rangle,$$

where $u_{ik}^{\backslash j} = \sum_{j'\neq j} z_{ij'}\beta_{j'k}$ is factor k constructed excluding the jth feature. It is easy to check that

$$\langle \gamma_{jk}\rangle = \omega_{jk},\ \langle \hat{\beta}_{jk}\rangle = \mu_{jk}\omega_{jk},\ \langle \hat{\beta}_{jk}^2\rangle = \left(\mu_{jk}^2 + \zeta_{jk}^2\right)\omega_{jk} + (1-\omega_{jk})\zeta_{0jk}^2,$$

$$\langle\beta_{jk}\rangle = \mu_{jk}\omega_{jk},\ \langle\beta_{jk}^2\rangle = \left(\mu_{jk}^2 + \zeta_{jk}^2\right)\omega_{jk}.$$

The variational parameters $(\mu_{jk}, \zeta_{jk}^2, \zeta_{0jk}^2)$ are chosen to maximize the ELBO, or equivalently, maximize

$$L(\beta_{jk}) = L_0(\beta_{jk}) + L_1(\beta_{jk}) - L_2(\beta_{jk}).$$

Omitting the part irrelevant to β_{jk}, and letting $A_1 = \sum_i (I_{i1} - I_{i2})z_{ij}$, $A_2 = \sum_i I_{i3}z_{ij}^2$, we have

$$\begin{aligned} L(\beta_{jk}) = &\ \omega_{jk}\left\{A_1\mu_{jk} - \frac{1}{2}(A_2 + \langle\tau_{g_jk}\rangle)\left(\mu_{jk}^2 + \zeta_{jk}^2\right) + \frac{1}{2}\ln\zeta_{jk}^2\right\} \\ &+ (1-\omega_{jk})\left\{-\frac{1}{2}\langle\tau_{g_jk}\rangle\zeta_{0jk}^2 + \frac{1}{2}\ln\zeta_{0jk}^2\right\} \\ &+ \omega_{jk}\langle\ln\frac{\pi_{g_jk}}{1-\pi_{g_jk}}\rangle - \omega_{jk}\ln\frac{\omega_{jk}}{1-\omega_{jk}} - \ln(1-\omega_{jk}) \end{aligned} \tag{10}$$

- μ_{jk} is chosen as

$$\mu_{jk} = \arg\max\left(A_1\mu_{ij} - \frac{1}{2}A_2\mu_{ij}^2 - \frac{\langle\tau_{g_jk}\rangle}{2}\mu_{jk}^2\right) = \frac{A_1}{\langle\tau_{g_jk}\rangle + A_2}.$$

- ζ_{jk}^2 and ζ_{0jk}^2 are chosen as

$$\begin{aligned} \zeta_{jk}^2 &= \arg\max\left(-\frac{1}{2}A_2\zeta_{jk}^2 - \frac{\langle\tau_{g_jk}\rangle}{2} + \frac{1}{2}\ln\zeta_{jk}^2\right) = \frac{1}{\langle\tau_{g_jk}\rangle + A_2}, \\ \zeta_{0jk}^2 &= \arg\max\left(-\frac{\langle\tau_{g_jk}\rangle}{2} + \frac{1}{2}\ln\zeta_{jk}^2\right) = \frac{1}{\langle\tau_{g_jk}\rangle}. \end{aligned}$$

- ω_{jk} is chosen as

$$\begin{aligned} \omega_{jk} &= \arg\max\left(\omega_{jk}\left(\frac{\mu_{jk}^2}{2\zeta_{jk}^2} + \frac{1}{2}\ln\frac{\zeta_{jk}^2}{\zeta_{0jk}^2} + \ln\langle\frac{\pi_{g_jk}}{1-\pi_{g_jk}}\rangle\right) - \omega_{jk}\ln\omega_{jk} - (1-\omega_{jk})\log(1-\omega_{jk})\right) \\ &= \frac{\exp(\lambda_{jk})}{1+\exp(\lambda_{jk})} \end{aligned}$$

  where $\lambda_{jk} = \frac{\mu_{jk}^2}{2\zeta_{jk}^2} + \frac{1}{2}\ln\frac{\zeta_{jk}^2}{\zeta_{0jk}^2} + \ln\langle\frac{\pi_{g_jk}}{1-\pi_{g_jk}}\rangle$.

Update $q(B_{jk})$. The expression of the expected log prior $L_0(B_{jk})$ and the expected log variational distribution $L_2(B_{jk})$ is similar to that of $L_0(\beta_{jk})$ and $L_2(\beta_{jk})$, except for replacing all quantities by their corresponding component for B_{jk}. The likelihood related part is

$$L_1(B_{jk}) \propto -\frac{1}{2}w_j\langle\nu_j\rangle\langle\|\mathbf{X}_j - \mathbf{U}\mathbf{B}_j\|_2^2\rangle. \tag{11}$$

Rearrange the terms, we have

$$L_1(B_{jk}) \propto w_j\langle\nu_j\rangle\left(\langle\mathbf{X}_j - \mathbf{U}_{k^c}\mathbf{B}_{k^c,j}\rangle^{\perp}\langle\mathbf{U}_k\rangle\langle B_{jk}\rangle - \frac{1}{2}\langle\|\mathbf{U}_k\|_2^2\rangle\langle B_{jk}^2\rangle\right).$$

We set $A_1 = w_j\langle\nu_j\rangle\,\langle\mathbf{X}_j - \mathbf{U}_{k^c}\mathbf{B}_{k^c,j}\rangle^\perp\,\langle\mathbf{U}_k\rangle$ and $A_2 = w_j\langle\nu_j\rangle\langle\|\mathbf{U}_k\|_2^2\rangle$, then,

$$L(B_{jk}) = \tilde{\omega}_{jk}\left\{A_1\tilde{\mu}_{jk} - \frac{1}{2}(A_2 + \langle\tau_{g_jk}\rangle)\left(\tilde{\mu}_{jk}^2 + \tilde{\zeta}_{jk}^2\right) + \frac{1}{2}\ln\tilde{\zeta}_{jk}^2\right\} + (1-\tilde{\omega}_{jk})\left\{-\frac{1}{2}\langle\tau_{g_jk}\rangle\tilde{\zeta}_{0jk}^2 + \frac{1}{2}\ln\tilde{\zeta}_{0jk}^2\right\} + \tilde{\omega}_{jk}\langle\ln\frac{\pi_{g_jk}}{1-\pi_{g_jk}}\rangle - \tilde{\omega}_{jk}\ln\frac{\tilde{\omega}_{jk}}{1-\tilde{\omega}_{jk}} - \ln(1-\tilde{\omega}_{jk}) \tag{12}$$

Hence, our update is

$$\tilde{\zeta}_{jk}^2 = \frac{1}{A_2 + \langle\tau_{g_jk}\rangle},\ \tilde{\zeta}_{0jk} = \frac{1}{\langle\tau_{g_jk}\rangle},$$
$$\tilde{\mu}_{jk} = \frac{A_1}{A_2 + \langle\tau_{g_jk}\rangle},\ \tilde{\omega}_{jk} = \frac{\exp(\lambda_{jk})}{1+\exp(\lambda_{jk})},$$

where $\lambda_{jk} = \frac{\tilde{\mu}_{jk}^2}{2\tilde{\zeta}_{jk}^2} + \frac{1}{2}\ln\frac{\tilde{\zeta}_{jk}^2}{\tilde{\zeta}_{0jk}^2} + \ln\langle\frac{\pi_{g_jk}}{1-\pi_{g_jk}}\rangle$.

Update $q(\bar{B}_j)$. We set $A_1 = \langle\bar{\nu}_j\rangle\langle\mathbf{U}\rangle^\perp\,\langle\mathbf{Y}_j\rangle$ and $A_2 = \langle\bar{\nu}_j\rangle\langle\mathbf{U}^\perp\mathbf{U}\rangle + \Lambda$, where $\Lambda = diag\{\tau_0^2,\ldots,\tau_0^2\}$, the ELBO related to $q(\bar{B}_{jk})$ is

$$L(\overline{\mathbf{B}}_j) \propto A_1^\perp\langle\overline{\mathbf{B}}_j\rangle - \frac{1}{2}\langle\overline{\mathbf{B}}_j^\perp A_2\overline{\mathbf{B}}_j\rangle + \frac{1}{2}\sum_{k=1}^K\ln\bar{\zeta}_{jk}^2 = A_1^\perp\bar{\mu}_j - \frac{1}{2}\bar{\mu}_j^\top A_2\bar{\mu}_j - \frac{1}{2}\sum_{k=1}^K A_{2kk}\xi_{jk}^2 + \frac{1}{2}\sum_{k=1}^K\ln\bar{\zeta}_{jk}^2.$$

The optimal updating parameters is

$$\bar{\mu}_j = A_2^{-1}A_1,\ \bar{\zeta}_{jk}^2 = \frac{1}{A_{2jk}}.$$

In practice, we consider a constrained solution where we require $\|\bar{\mu}_j\|_2 \geq L_{\overline{B}_j}$. This extra constraint is to alleviate the extra volatility due to the oscillation between $(\overline{B}, \mathbf{B})$ and $\boldsymbol{\beta}$ when w, the weight on $\mathbf{X}$, is small. By default, we let $L_{\overline{B}_j} = (1-w)\vee 0$. This constraint does not affect $\bar{\zeta}_{jk}^2$, but we no longer has a closed form expression for $\bar{\mu}_j$. However, we can numerically find the optimal solution:

1. Consider the ridge penalized problem for $\bar{\mu}_j$:
$$\bar{\mu}_j(\alpha) = \arg\max_{\bar{\mu}_j} A_1^\top\bar{\mu}_j - \frac{1}{2}\bar{\mu}_j^\top(A_2 + \alpha\mathbf{Id})\bar{\mu}_j$$
2. Let α^* be the largest non-positive value such that $\|\bar{\mu}_j(\alpha^*)\|_2^2 \geq L_{\overline{B}_j}$, which can be found via binary search.
3. Then, $\bar{\mu}_j = \bar{\mu}_j(\alpha^*)$ solves the constrained problem (Guan, 2021; Guan, 2022).
$$\bar{\mu}_j(\alpha) = \arg\max_{\bar{\mu}_j:\|\bar{\mu}_j\|_2^2\geq L_{\overline{B}_j}} A_1^\top\bar{\mu}_j - \frac{1}{2}\bar{\mu}_j^\top A_2\bar{\mu}_j.$$

Update $q(\boldsymbol{\mathcal{E}})$**.** The expected logarithm from the ELBO calculation related to $\boldsymbol{\mathcal{E}}_{ik}$ is given below (used the fact that $\langle \boldsymbol{\mathcal{E}}_{ik} \rangle = 0$):

$$L(\boldsymbol{\mathcal{E}}_{ik}) \propto \frac{1}{2}\left(-\sum_{j=1}^{p} w_j \langle \nu_j \rangle \langle B_{ij}^2 \rangle e_{jk}^2 - \sum_{j=1}^{\overline{p}} \langle \bar{\nu}_j \rangle \langle \bar{B}_{ij}^2 \rangle e_{jk}^2 - \frac{e_{ik}^2}{2} + \ln e_{ik}^2 \right).$$

To avoid instability, we also require $e_{jk}^2 \geq C_{\boldsymbol{\mathcal{E}}}$ and $C_{\boldsymbol{\mathcal{E}}} = 0.1$ by default. Let $A = \frac{1}{\sum_{j=1}^{p} w_j \langle \nu_j \rangle \langle B_{jk}^2 \rangle + \sum_{j=1}^{\overline{p}} \langle \bar{\nu}_j \rangle \langle \bar{B}_{jk}^2 \rangle e_{ik}^2 + 1}$. The optimal solution under this constraint is

$$e_{jk}^2 = \begin{cases} A & \text{if } A \geq C_{\boldsymbol{\mathcal{E}}} \\ C_{\boldsymbol{\mathcal{E}}} & \text{otherwise} \end{cases}.$$

Update $q(\tau)$**.** The parameter τ appear only at the prior and the hyper prior, its related ELBO at parameter $\theta = (\tilde{a}_{gk}^1, \tilde{c}_{gk}^1)$ is

$$L(\tau_{gk}) \propto -\left(\frac{\sum_{g_j=g}\left(\langle \hat{\beta}_{jk}^2 \rangle + \langle \hat{B}_{jk}^2 \rangle\right)}{2} + c_1 - \tilde{c}_{gk}^1\right)\langle \tau_{gk} \rangle + \left(|\mathcal{G}_g| + a_1 - \tilde{a}_{gk}\right)\langle \ln \tau_{gk} \rangle + \ln \Gamma(\tilde{a}_{gk}^1, \tilde{c}_{gk}^1),$$

where $\mathcal{G}_g$ is the set of feature indices in group g and $|\mathcal{G}_g|$ is the set size. The optimal update rule is

$$\tilde{a}_{gk}^1 = a_0 + |\mathcal{G}_g|, \quad \tilde{c}_{gk}^1 = c_1 + \frac{\sum_{g_j=g}\left(\langle \hat{\beta}_{jk}^2 \rangle + \langle \hat{B}_{jk}^2 \rangle\right)}{2}.$$

Intuitively, when $\langle \tau_{gk} \rangle = \frac{\tilde{a}_{gk}^1}{\tilde{b}_{gk}}$ is large, we will have a prior more concentrated around 0, and hence, a denser model with small effects. When $\langle \tau_{gk} \rangle$ is very small, on the other hand, we will only let a feature being non-zero if it's effect size is very large. As a result, the model does not favor $\langle \tau_{gk} \rangle$ being too small or too large. Since $\tilde{a}_{gk}^1 = a_0 + |\mathcal{G}_g|$ is fixed, if we want $\langle \tau_{gk} \rangle \in [L_{low}, L_{up}]$, we can restrict $\tilde{c}_g^1$ to be in the range $[\frac{\tilde{a}_{gk}^1}{L_{up}}, \frac{\tilde{a}_{gk}^1}{L_{low}}]$. By default, we let $L_{low} = \frac{\ln p}{n}$ and $L_{up} = 1$.

Update $q(\pi)$**.** The ELBO related to π_{gk} at the variational parameter $\theta = (\tilde{a}_{gk}^2, \tilde{c}_{gk}^2)$ is

$$L(\pi_{gk}) \propto \left(a_2 + \sum_{g_j=g}\left(\langle \gamma_{jk} \rangle + \langle s_{jk} \rangle\right) - \tilde{a}_{gk}^2\right)\langle \ln \pi_{gk} \rangle + \left(c + \sum_{g_j=g}\left(2 - \langle \gamma_{jk} \rangle - \langle s_{jk} \rangle\right) - \tilde{c}_{gk}^2\right)\langle \ln(1-\pi_{gk}) \rangle + \ln \mathrm{Beta}(\tilde{a}_{gk}^2, \tilde{c}_{gk}^2).$$

The optimal solution is

$$\tilde{a}_{gk}^2 = a_2 + \sum_{g_j=g}\left(\langle \gamma_{jk} \rangle + \langle s_{jk} \rangle\right), \ \tilde{c}_{gk}^2 = c + \sum_{g_j=g}\left(2 - \langle \gamma_{jk} \rangle - \langle s_{jk} \rangle\right).$$

The average sparsity level is $\langle \pi_{gk} \rangle = \frac{\tilde{a}_{gk}^2}{\tilde{a}_{gk}^2 + \tilde{c}_{gk}^2} = \frac{\tilde{a}_{gk}^2}{a_2 + c_2 + 2|\mathcal{G}_g|}$. We can also have a user specified upper bound on this average sparsity level. Let α be the specified sparsity

level upper bound with a default value at 0.5. We find the optimal solution under the constraint

$$\tilde{c}^2_{gk} + \tilde{a}^2_{gk} = a_2 + c_2 + 2|\mathcal{G}_g|, \tilde{a}^2_{gk} \leq \alpha \left(a_2 + c_2 + 2|\mathcal{G}_g|\right).$$

If the optimal solution already satisfies the constraint, we don't need any modifications; otherwise, we let

$$\tilde{a}^2_{gk} = \alpha \left(a_2 + c_2 + 2|\mathcal{G}_g|\right), \ \tilde{a}^2_{gk} = (1-\alpha) \left(a_2 + c_2 + 2|\mathcal{G}_g|\right).$$

Update $q(\nu)$ and $q(\bar{\nu})$. Let $A_1 = w_j \left(\frac{N_j}{2} + a_0\right)$, $A_2 = w_j \left(\frac{\langle \|\mathbf{X}_j - \mathbf{U}\mathbf{B}_k\|_2^2\rangle}{2} + c_0\right)$, the ELBO related to ν_j is the converse variance of $\mathbf{X}_j$

$$L(\nu_j) \propto \langle \ln \Gamma(\nu_j|A_1, A_2)\rangle - \langle \ln \Gamma(\nu_j|\tilde{a}^X_{j0}, \tilde{c}^X_{j0})\rangle.$$

The optimal solution is

$$\tilde{a}^X_{j0} = w_j \left(\frac{N_j}{2} + a_0\right), \ \tilde{c}^X_{j0} = w_j \left(\frac{\langle \|\mathbf{X}_j - \mathbf{U}\mathbf{B}_j\|_2^2\rangle}{2} + c_0\right).$$

Let $A_1 = \left(\frac{N_j}{2} + a_0\right)$, $A_2 = \left(\frac{\langle \|\mathbf{Y}_j - \mathbf{U}\mathbf{B}_k\|_2^2\rangle}{2} + c_0\right)$, the ELBO related to $\bar{\nu}_j$ is the converse variance of $\mathbf{Y}_j$

$$L(\bar{\nu}_j) \propto \langle \ln \Gamma(\bar{\nu}_j|A_1, A_2)\rangle - \langle \ln \Gamma(\bar{\nu}_j|\tilde{a}^Y_{j0}, \tilde{c}^Y_{j0})\rangle.$$

The optimal solution is

$$\tilde{a}^Y_{j0} = \left(\frac{N_j}{2} + a_0\right), \ \tilde{c}^Y_{j0} = \left(\frac{\langle \|\mathbf{Y}_j - \mathbf{U}\mathbf{B}_j\|_2^2\rangle}{2} + c_0\right).$$

ELBO increase. We can keep track of the ELBO increase by adding up the ELBO increase in each updating step. For given parameters, e.g., β_{jk}, let $L_{old}(.)$ be the values from using the variational parameters that are currently under investigation, and let $L(.)$ be the evaluation updated parameters. We can calculate the related ELBO increase as follows:

$$\Delta(\beta_{jk}) = L(\beta_{jk}) - L_{old}(\beta_{jk}),$$

where $L(\beta_{jk})$ is the ELBO related to β_{jk} using θ and $L_{old}(\beta_{jk})$ is the ELBO related to β_{jk} using θ^{old}. The total increase in ELBO can be calculated as

$$\Delta = \sum_{k=1}^K \sum_{j=1}^p \Delta(\beta_{jk}) + \sum_{k=1}^K \sum_{j=1}^p \Delta(B_{jk}) + \sum_{j=1}^{\bar{p}} \Delta(\bar{B}_j) + \sum_{j=1}^{p} \Delta(\nu_j) + \sum_{j=1}^{\bar{p}} \Delta(\bar{\nu}_j)$$
$$+ \sum_{k=1}^K \sum_{g=1}^G \left(\Delta(\tau_{gk}) + \Delta(\pi_{gk})\right).$$

C. Parameter estimation with non-Gaussian response

Currently, SPEAR also supports two-class or multi-class classifications and ordinal regression. For these response types, the evidence lower bound under the given variational distribution becomes intractable. There are usually two ways for variational

parameter updates when the expectation is intractable: (1) find a lower bound of the ELBO that has closed form and optimize for this lower bound, or (2) use a numerical estimate of the gradient and update using stochastic gradient descent. There are pros and cons for both methods. For the former, we no longer optimize with respect to the original objective. For the later, there is variability of the derivative as well as the objective value due to the stochastic nature and can incur a large computational cost in order to have low variability. Here, we consider the approach of using the lower bound. The lower bounds for all three data types are based on results from Jordan et al. [1999]. The bound from Jordan et al. [1999] is used for the logistic model, and an variant is used for bounding the multinomial logistic regression loss Bouchard [2007]. We further extend this result to ordinal regression in this paper.

C.1. Lower Bounds of ELBO

If the expectation of the log-likelihood under the variational distribution is intractable, one popular way to circumvent it is to consider a good lower bound of this expectation that has a nice form with the help from extra augmenting variables and optimize in the enlarged parameter space. In general, let $f(\theta)$ be the log likelihood function. If its expectation is intractable under the variational distributions, but the quadratic function of θ can be explicitly calculated. Then, if there exist functions $a(\xi)$ and $A(\xi)$ for the augmenting variable ξ, such that

$$f(\theta) \geq a(\xi)^T\theta + \theta^T A(\xi)\theta + C(\xi) := g(\xi, \theta)$$

We have $f(\theta) \geq \max_\xi[f(\xi) + a(\xi)^T\theta + \theta^T A(\xi)\theta + C(\xi)]$, and

$$E_q f(\theta) \geq \max_\xi [C(\xi) + E_q\theta^T A(\xi)\theta + E_q(a(\xi)^T\theta)].$$

Hence, we can optimize for q given ξ, and optimize for ξ given q. This guarantees that the augmented lower-bound function $E_q g(\xi, \theta)$ is non-decreasing in the updating steps. We will consider two particular response types: logistic regression (two classes or multiple classes) and ordinal regression. The key results used to derive these lower bounds are from Jordan et al. [1999]: for any η, let $f(\eta) = \frac{1}{\exp(-\eta)+1}$ and $\lambda(\eta) = \tanh(\frac{\eta}{2})/(4\eta)$, then, we have

$$f(\eta) \geq f(\xi)\exp\left(\frac{\eta - \xi}{2} - \lambda(\xi)(\eta^2 - \xi^2)\right), \ \forall \xi. \tag{13}$$

A slightly more relaxed bound is

$$f(\eta) \geq f(\xi)\exp\left(\frac{\eta - \xi}{2} - \lambda(\xi)(\eta^2 - \xi^2) - \epsilon\eta^2\right), \ \forall \xi, \forall \epsilon \geq 0 \tag{14}$$

We let $\epsilon \geq 0$ be a user specified small constant, and it can encourage more numerical stability in the case of perfect separation.

C.2. Two-class logistic regression

Let $\eta_i = (\mathbf{u}_i^T \mathbf{B}_j + \alpha)$ be our link function value for response j and sample i in the two-class logistic model, and α is the intercept. Let $H_i = (2S_i - 1)\eta_i$ where $S_i \in \{0, 1\}$ indicates the class label. The expected log likelihood of sample i, $\langle \ell_i \rangle = \langle \log \frac{1}{1+\exp(\eta_i)} \rangle$, can be lower bounded with Eq. (14):

$$\langle \ell_i \rangle \geq \max_{\xi_i} \frac{\langle H_i \rangle - \xi_i}{2} - \lambda(\xi_i)(\langle H_i^2 \rangle - \xi_i^2) - \epsilon \langle H_i^2 \rangle.$$

Following the same argument as in Jordan et al. [1999], despite the additional term ϵ, the choice of ξ for maximizing the lower bound given the variational distribution is:

$$\xi_i = \sqrt{\langle H_i^2 \rangle} = \sqrt{\langle \eta_i^2 \rangle}. \tag{15}$$

We then approximate the lower bound $\langle \ell_i \rangle$ with the current ξ_i given by Eq. (15), and we update the variational distribution using this lower bound:

$$\underline{\ell}_i = -\left(\lambda(\xi_i) + \epsilon\right) \left\{ \left(\eta_i - \frac{(2S_i - 1)}{4(\lambda(\xi_i) + \epsilon)} \right)^2 \right\} \tag{16}$$

In other words, we are equivalently considering the following response when updating the variational distribution:

$$\underline{Y}_i \sim \mathcal{N}\left(\frac{(S_i - \frac{1}{2})}{2(\lambda(\xi_i) + \epsilon)} - \alpha, \frac{1}{2(\lambda(\xi_i) + \epsilon)}\right).$$

where the coefficient α can be first estimated by

$$\alpha = \frac{\sum_i \left((\lambda(\xi_i) + \epsilon) \left(\underline{Y}_i - \langle \mathbf{u}_i^T \mathbf{B}_j \rangle \right) \right)}{2 \sum_i \left(\lambda(\xi_i) + \epsilon \right)}.$$

C.3. Ordinal logistic regression

Suppose that we are looking at the response Y_i that takes value in $\{1, \ldots, M\}$. 1 is the base class and we are interested in estimation $P(Y_i \geq m)$ for $m = 2, \ldots, M$. Let $\eta_i = \mathbf{u}_i^T \mathbf{B}_j$ and $\infty = \alpha_1 > \ldots > \alpha_K > \alpha_{K+1} = -\infty$. The probability for observing a label at least k is modeled as $P(Y_i \geq m) = \frac{1}{1+\exp(-\eta_i - \alpha_m)}$. The log-likelihood contributed by sample i is

$$\ell_i = \sum_{m=1}^{M} \mathbb{1}(Y_i = m) \log \left(P(Y_i \geq m) - P(Y_i \geq m+1) \right).$$

After some simple rearrangements, we have

$$\ell_i = \begin{cases} -\eta_i - \alpha_2 - \log(1 + \exp(-\eta_i - \alpha_2)) & \text{if } Y_i = 1 \\ -\log(1 + \exp(-\eta_i - \alpha_M)) & \text{if } Y_i = M \\ -\eta_i + \log(\exp(-\alpha_{y_i+1}) - \exp(-\alpha_{y_i})) - \log(1 + \exp(-\eta_i - \alpha_{y_i})) - \log(1 + \exp(-\eta_i - \alpha_{y_i+1})) & \text{otherwise} \end{cases}.$$

We now apply Eq. (14) again to each of the terms, and introduce auxiliary variables ξ_{im} for $m = 2, \ldots, M$. The lower bound of the expected log likelihood given ξ_{im} and α_m is then:

$$\langle \underline{\ell}_i \rangle = \begin{cases} \langle -\frac{\eta_i}{2} - \left(\lambda(\xi_{i,2}) + \epsilon\right) \left(\eta_i^2 + 2\alpha_2 \eta_i\right) \rangle & \text{if } Y_i = 1 \\ \langle \frac{\eta_i}{2} - \left(\lambda(\xi_{iM}) + \epsilon\right) \left(\eta_i^2 + 2\alpha_M \eta_i\right) \rangle & \text{if } Y_i = M \\ \langle -\left(\lambda(\xi_{i,Y_i}) + \epsilon\right) \left(\eta_i^2 + 2\alpha_{Y_i} \eta_i\right) - \left(\lambda(\xi_{i,Y_i+1}) + \epsilon\right) \left(\eta_i^2 + 2\alpha_{Y_i+1} \eta_i\right) \rangle & \text{otherwise} \end{cases}$$

As a result, we replace Y_i with the transformed response

$$\underline{Y}_i \sim \begin{cases} \mathcal{N}\left(-\frac{1}{4(\lambda(\xi_{i2})+\epsilon)} - \alpha_2, \frac{1}{2(\lambda(\xi_{i2})+\epsilon)} \right) & \text{if } Y_i = 1 \\ \mathcal{N}\left(-\frac{1}{4(\lambda(\xi_{iM})+\epsilon)} - \alpha_M, \frac{1}{2(\lambda(\xi_{iM})+\epsilon)} \right) & \text{if } Y_i = M \\ \mathcal{N}\left(\frac{\alpha_{y_i}\left(\lambda(\xi_{i,y_i})+\epsilon\right) + \alpha_{y_i+1}\left(\lambda(\xi_{i,y_i+1})+\epsilon\right)}{\left(\lambda(\xi_{i,y_i})+\epsilon\right) + \left(\lambda(\xi_{i,y_i+1})+\epsilon\right)}, \frac{1}{2[\left(\lambda(\xi_{i,y_i})+\epsilon\right) + \left(\lambda(\xi_{i,y_i+1})+\epsilon\right)]} \right) & \text{otherwise} \end{cases}.$$

Update of ξ_{im}: Following the same argument as in Jordan et al. [1999], we have $\xi_{im} = \sqrt{\langle(\eta_i + \alpha_m)^2\rangle}$ to achieve the tightest bound.
Update of α_m: Given other parameters, we can update the intercepts terms $\infty = \alpha_1 > \alpha_2, \ldots, \alpha_M > \alpha_{M+1} = -\infty$ to maximize the lower bound with some general optimizer (to omit constants). For simplicity, we have let $\lambda(\xi_{im}) \leftarrow \lambda(\xi_{im}) + \epsilon$ in the calculation below:

$$\sum_i \underline{\ell}_i = \sum_{y_i=1}\{-\frac{\alpha_2}{2} - \lambda(\xi_{i,1})(\alpha_2^2 + 2\eta_i\alpha_2)\} + \sum_{Y_i=M}\{\frac{\alpha_M}{2} - \lambda(\xi_{iM})(\alpha_M^2 + 2\eta_i\alpha_M)\}$$
$$+ \sum_{Y_i=2}^{M-1}\left\{\log(1-\exp(-\alpha_{Y_i} + \alpha_{Y_i+1})) + \frac{\alpha_{y_i}}{2} - \frac{\alpha_{y_i+1}}{2} - \lambda(\xi_{i,Y_i})(\alpha_{y_i}^2 + 2\eta_i\alpha_{y_i}) - \lambda(\xi_{i,Y_i+1})(\alpha_{Y_i+1}^2 + 2\eta_i\alpha_{Y_i+1})\right\}$$

Let $\Delta_m = \alpha_{m+1} - \alpha_m > 0$. Then, we want to minimize the neg-loglikelihood:

$$A_1\alpha_2^2 + B_1\alpha_2 + \sum_{m=2}^{M}\{B_k(\alpha_2 + \sum_{l=2}^{m-1}\Delta_l) + A_k(\alpha_1 + \sum_{l}^{m-1}\Delta_l)^2\} + \sum_{m=2}^{M-1}N_m\{\log(1-\exp(-\Delta_m))\}$$

where for $m = 1, \ldots, K$, we define $n_m = |\{i : y_i = m\}|$ and

$$A_m = -\sum_{Y_i=m}\lambda(\xi_{im}) - \sum_{Y_i=(m-1)}\lambda(\xi_{im}),$$
$$B_m = \frac{n_m - n_{m-1}}{2} - 2\sum_{Y_i=m-1}\lambda(\xi_{i,Y_i+1})\eta_i - 2\sum_{Y_i=k}\lambda(\xi_{i,Y_i})\eta_i.$$

We can solve for α_2 and Δ_2, ..., Δ_{M-1} under the constraint that $\Delta_m > 0$.

C.4. Multi-class logistic regression

A third common response type is the multi-label response. SPEAR models this response type with a multi-class logistic regression problem:

$$\ell_i = \sum_{m=1}^{M}\mathbb{1}(Y_i = m)\log P_m(x_i|\theta, \alpha),$$

where $P_m(x_i|\theta, \alpha) = \frac{\exp(\theta_m^T x_i + \alpha_m)}{\sum_{m'}\exp(\theta_{m'}^T x_i + \alpha_{m'})}$. As a result, when $Y_i = m$, we have

$$\ell_i = \theta_m^T x_i + \alpha_m - \log(\sum_{m'=1}^{M}\exp(\theta_{m'}^T x_i + \alpha_{m'})).$$

As before, to have a closed form integral of the variational distribution, we are going to upper bound the log of the exponential sums with a quadratic function and thus provides a lower bound the log-likelihood. Generalization of Jordan et al. [1999] to multi-class logistic regression has been studied before in Bouchard [2007]:

$$\log\sum_{m=1}^{M}\exp(\eta_{im}) = \zeta_i + \log\sum_{m=1}^{M}\exp(\eta_{im} - \zeta_i)$$

$$\leq \zeta_i + \sum_{m=1}^{M} \log(1 + \exp(\eta_{im} - \zeta_i))$$

$$\leq \zeta_i + \sum_{m=1}^{M} \left\{ \frac{\eta_{im} - \zeta_i - \xi_{im}}{2} + \lambda(\zeta_m)((\eta_{im} - \zeta_i)^2 - \xi_{im}^2) + \epsilon \eta_{im}^2 + \log(1 + \exp(\xi_{im})) \right\}$$

The optimal expressions for ξ_{im} and ξ take the following bound:

$$\xi_{im}^2 = \langle (\eta_{im} - \zeta_i)^2 \rangle = \langle \eta_{im}^2 \rangle + \zeta_i^2 - 2\zeta_i \langle \eta_{im} \rangle$$

$$\zeta_i = \frac{\frac{1}{2}(\frac{M}{2} - 1) + \sum_{m=1}^{M} (\lambda(\xi_{im}) + \epsilon) \langle \eta_{im} \rangle}{\sum_{m=1}^{M} (\lambda(\xi_{im}) + \epsilon)}$$

As a result, given ξ_{im}, ζ_i and the intercepts α_m, we can consider a normal response

$$\underline{Y}_{im} \sim \mathcal{N}\left(\frac{\mathbb{1}_{Y_{im}=1} - \frac{1}{2} - 2\left(\lambda(\xi_{im}) + \epsilon\right)\zeta_i}{2\left(\lambda(\xi_{im}) + \epsilon\right)} - \alpha_m, \frac{1}{2\left(\lambda(\xi_{im}) + \epsilon\right)}\right),$$

where the intercepts α_m are estimated as

$$\alpha_m = \frac{\sum_i \left((\lambda(\xi_{im}) + \epsilon) \left(\underline{Y}_{im} - \langle \mathbf{u}_i^T \mathbf{B}_j \rangle \right) \right)}{2 \sum_i (\lambda(\xi_{im}) + \epsilon)}.$$

D. Additional results on synthetic data

D.1. Gaussian Simulation

Gaussian Simulation Overview

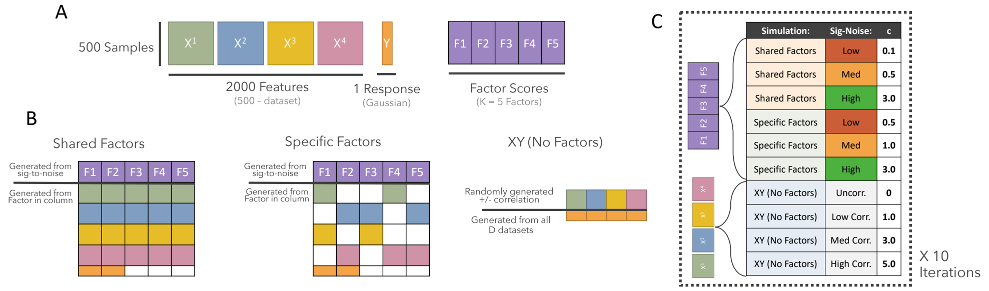

(A) Dimensions of the simulated multi-omic datasets, the Gaussian response of interest and the five simulated factor scores. (B) Representation of the three different types of scenarios: shared factors, specific factors, and no factors. (C) table of each set of parameters tested in the total Gaussian simulation across scenarios, signal-to-noises, and feature correlation.

We simulated synthetic multi-omic data in three distinct scenarios:

1. Shared factors: 5 true factors were generated (U), all of which are used to generate X (first 2 factors used to generate Y)

2. Specific factors: 5 true factors were generated (U), each one randomly influences 2 datasets in X (first 2 factors used to generate Y)

3. No factors (XY): X is generated randomly (with varying levels of correlation amongst the features), and Y is randomly generated directly from X

In each scenario, X was generated for 500 training samples and 2000 testing samples. We simulated X to contain 4 synthetic multi-omics assays, each with 500 synthetic analytes (for a total of 2000 synthetic analytes across the 4 assays). Each combination of parameters was repeated 10 times across a grid of signal-to-noise ratios (defined below).

The Gaussian scenario when factors were simulated (shared factors and specific factors) was performed in the following manner:

1. Let $\mathbf{U}$ represent the simulated factor scores with dimensions $k \times N$ and let $\mathbf{B}$ represent the simulated projection coefficients with dimensions $p \times K$, where N is the number of simulated samples, p is the total number of simulated analytes, K is the total number of simulated factors, and c_1 is the modulated signal-to-noise coefficient. c takes a grid of values from 0.01 to 5. In particular, we pick $c_1 \in (0.5, 1.0, 3.0)$ as low, moderate, and high signal. $\mathbf{B}$ and $\mathbf{U}$ are simulated as follows:

$$B_{j,k} = N(0,1) \times c$$

$$U_{k,n} = N(0,1)$$

$$j = 1...p \quad k = 1...K \quad n = 1...N$$

2. Implement sparsity in $\mathbf{B}$ by nullifying any coefficients corresponding to factors that were not designated as influential to X. U_{XY} was defined by factors 1 and 2, whereas U_X was defined by all five factors.

3. X was generated in the following manner. Let E represent a matrix with the same dimensions as X ($N \times P$) filled with noise drawn from a normal distribution ($\mu = 0$, $\sigma^2 = 1$).

$$X = U_{XY}B + E \quad E \sim N(0,1)$$

4. Y was generated in a similar fashion. Let c_2 represent another sparsity coefficient used to control the spread of Y. We kept this parameter consistent at 1 for all our Gaussian simulations. Let 1 represent a vector of all ones utilized to take the row sums, and let K_Y represent the number of factors meant to influence Y, which we set to 2.

$$Y = 1^T U_Y \times \sqrt{\frac{c_2}{K_Y}} + E \quad E \sim N(0,1)$$

When simulating data without underlying factors, c_1 modifies the correlation amongst the features in X rather than noise in the factors (U). In these scenarios, we follow the entire aforementioned procedure with the exception of generating Y as a product of concatenated X and a new vector B of length p.

Results for "specific factors" are shown in the main paper. Results for "shared factors" followed the same trends as the "specific factors", only differing in the values of c_1 for low, moderate, and high signals. Results for "no factors (XY)" are shown in Supplementary Figure 1.

Supplementary Figure 1: No Factors (XY) Scenario Results

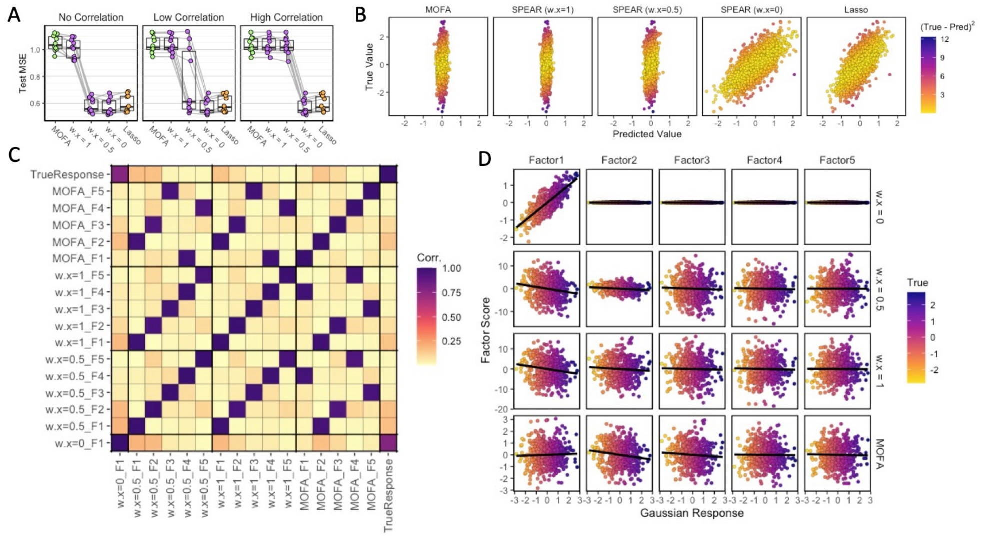

(A) Boxplots of mean-squared errors of the models on the testing data. Points are connected if they used the same simulated data from the same iteration. Results are shown for varying feature correlations, including no correlation, low correlation, and high correlation. (B) Scatterplots of true Gaussian response (y-axis) vs. predicted response (x-axis) for each model. Samples are colored by mean squared error. (C) Correlation heatmap between factor scores for each model and true response. (D) Scatterplots of various factor scores (y-axis) against the true Gaussian response (x-axis). Samples are colored by true Gaussian response.

By iterating over a gradient of signal-to-noise ratios, we found a consistent pattern of MOFA performing much worse than SPEAR with high w at a moderate signal-to-noise (1) (Supplemental Fig. 2). This is likely attributed to the lack of supervision in the MOFA model, as further inspection into the SPEAR and MOFA factors (Fig. 2b, Fig. 2c) revealed that MOFA was only able to construct the non-predictive factors in the data.

Supplementary Figure 2: All Specific Factors Gaussian Results

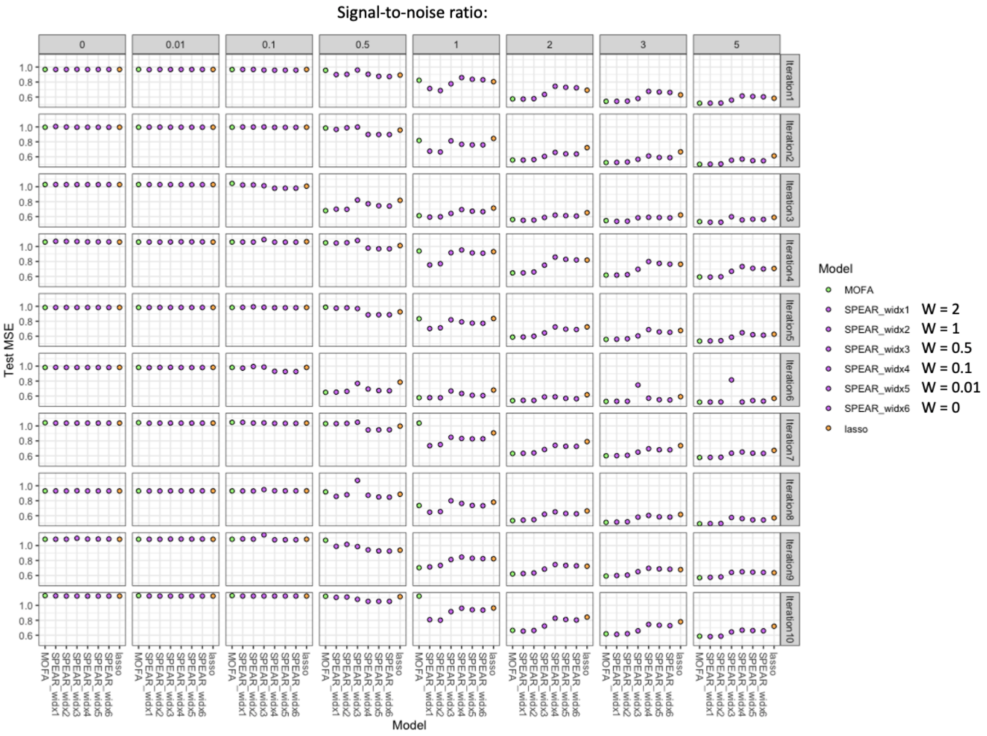

(A) Results for each iteration of the Gaussian simulation. Each model (x-axis) is presented with the resulting MSE on the testing cohort for all ten iterations (y-axis facet) and for all signal-to-noise ratios (x-axis facet).

D.2. Ordinal Simulation

To demonstrate SPEAR's ability to model different types of responses, we also carried out additional non-gaussian simulations. Like the Gaussian simulation, five true factors (U) were used to construct four multi-omics assays (X) and a non-Gaussian response (Y) for both training and testing datasets. In the ordinal simulation, the data and response were simulated as follows:

1. Randomly assign 500 training samples and 1000 testing samples a class from $1-7$
2. Assign class means (μ_1–μ_7) to be located at the following values: $(-1.5, -1.3, -1, 0, 1, 1.3, 1.5)$. The goal is to generate ordinal class signals that have a nonlinear trend.
3. Generate $U_{\|}$ as the first two factors in U: $(U_{\|} = \mu_{c_1} + N(500) \times \sqrt{\frac{1}{c_1}})$. c_1 was modulated to simulate various signal-to-noise ratios. The moderate case shown in Supplementary Figure 3 used $c_1 = 11$. The final term is to add noise to the model.
4. Finally, $U_{\perp}$ was generated as the other 3 factors in U randomly ($U_{\perp 1-3} = N(500)$)

5. X was then generated exactly the same way from the Gaussian simulation as described in the manuscript.

We then trained multiple SPEAR models with each treating the response differently (Gaussian, multinomial, and ordinal). In the moderate signal-to-noise, the moderate signal case demonstrated Gaussian SPEAR's difficulty handling the nonlinearity of the class signals in a linear representation (Fig. 3c), further seen in the Gaussian model's predictions (Fig. 3d). SPEAR using multinomial and ordinal responses achieved lower balanced misclassification errors, with the ordinal model outperforming the others. Ordinal SPEAR also extracted predictive signals capable of identifying all seven classes as represented by the correct median probabilities (Fig. 3e).

Supplementary Figure 3: Ordinal Simulation Results

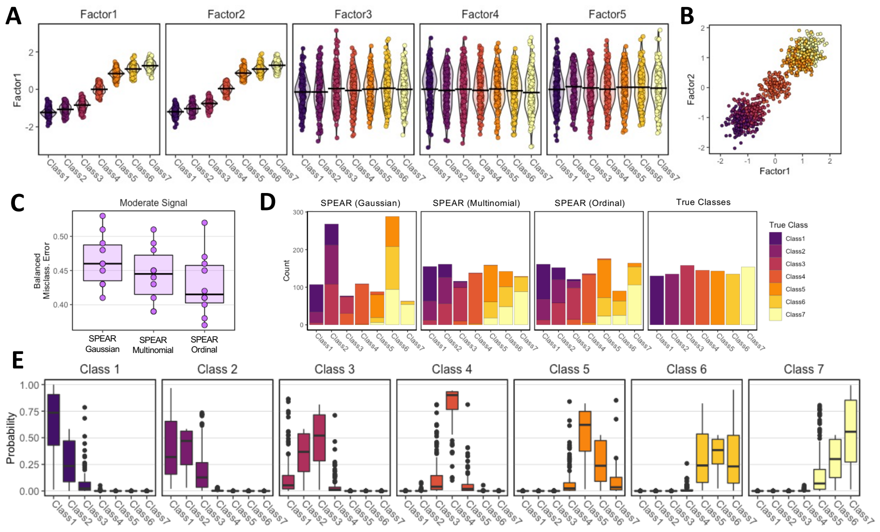

(A) Grouped violin plots of the simulated samples by true factor scores (y-axis) and their ordinal response class (x-axis). (B) Scatter plot of simulated samples by true factor 1 score (x-axis) and true factor 2 score (y-axis). (C) Grouped boxplots of balanced misclassification error results from SPEAR models on simulated test data. SPEAR models are differentiated by how the response was treated. (D) Histogram of simulated test data ordinal class predictions. Plots are sorted by predicted class (x-axis) and colored by true class. (E) Boxplots of ordinal class assignment probabilities derived from the SPEAR ordinal model. Samples are separated by their true ordinal class.

D.3. Multinomial Simulation

The multinomial simulation was carried out similarly to the Gaussian and ordinal simulations, with varying signal levels to simulate low, moderate, and high signal. Results for the moderate signal are shown below.

Multinomial response Y was simulated in the same manner as the ordinal section above, but with $U_{\|1}$ and $U_{\|2}$ being simulated separately. This is evident in Supplemental Figure 4B below, where certain classes were only distinguishable via either Factor 1 or Factor 2.

Supplementary Figure 4: Multinomial Simulation Results

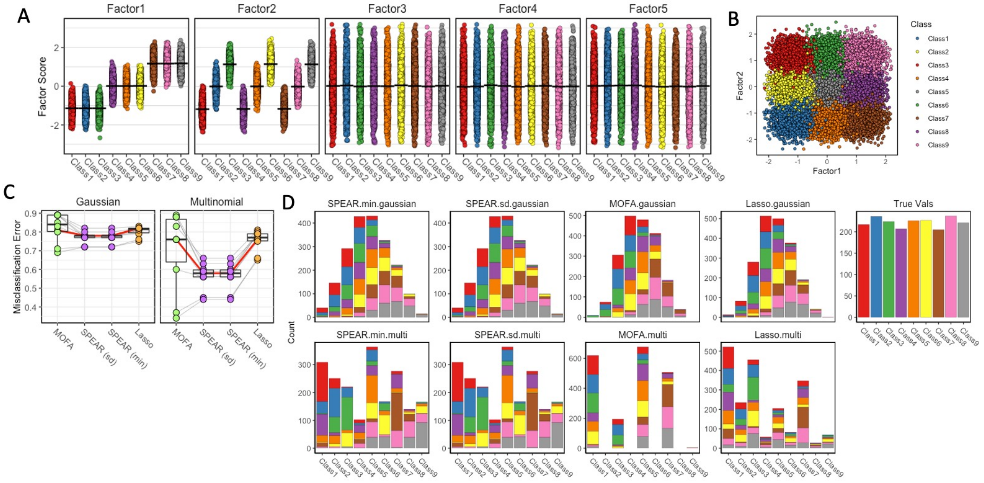

(A) Grouped violin plots of the simulated samples by true factor scores (y-axis) and their multinomial response class (x-axis). Factors 1 and 2 contain all the information necessary to discriminate classes. (B) Scatter plot of simulated samples by true Factor 1 score (x-axis) and true Factor 2 score (y-axis), colored by true multinomial class. (C) Grouped boxplots of balanced misclassification error results from various models on simulated test data. Gaussian models treated the response as a Gaussian variable, and multinomial models treated the response as multinomial. (D) Histograms of predicted classes from various models. Samples are colored by their true class.

E. Additional results on real data

E.1. TCGA-BC dataset background and preprocessing

The TCGA-BC dataset was adapted from Singh et al., consisting of 16851 mRNAs, 349 miRNAs, and 9482 CpG methylation sites. Each sample was taken from a primary solid breast cancer tumor that has been classified according to the PAM50 subtype signature, a 50-gene signature. The PAM50 signature is one of the main intrinsic breast cancer signatures used in clinical practice to determine the course of therapy (Wallden et al.; Goldhirsch et al.). Singh et al. showed that even when the 50 genes were removed from the dataset, it was still possible to predict subtype class using the remaining multi-omics features, indicating that there are underlying biological pathways that can distinguish and drive the subtypes.

While most preprocessing was kept as described in Singh et al., including the removal of the PAM50 signature mRNA genes, we additionally removed the least 20% variable features from each assay to reduce the total number of features. We did not exclude the PAM50 genes from the methylation results as the markers are used in gene expression

assays. Samples were then split into train ($n_{train} = 379$) and test ($n_{test} = 610$) sets as described in Singh et al. Finally, all three assays were scaled and centered to be normally distributed ($\mu = 0$, $\sigma^2 = 1$).

E.2. COVID-19 dataset background and preprocessing

The SARS-CoV-2 (COVID-19) dataset contains multi-omics data from 254 samples from SARS-CoV-2 positive patients and 124 samples from matched healthy subjects from Su et al.. Samples were taken from one of two timepoints, T1 and T2. At each timepoint, participants were assigned a COVID-19 severity score based on a World Health Organization (WHO) ordinal scale for clinical improvement: uninfected (0), ambulatory without (1) and with activity limitation (2), hospitalized without (3) and with oxygen therapy (4), hospitalized with non-invasive ventilation (5), intubation (6) or ventilation with additional organ support (7) and death (8). These ordinal values were condensed into four classes: healthy (0), mild (1-2), moderate (3-4), and severe (5-7).

MissForest, a non-parametric missing value imputation for mixed-type data was utilized to address missing values in both the proteomics and metabolomics (Stekhoven and Bühlmann). The metabolite expression values required quantile normalization and log transformation. Both proteomic and metabolomic expression values were then scaled and centered to be normally distributed ($\mu = 0$, $\sigma^2 = 1$).

E.3. SPEAR Factor Influence on Prediction

To investigate the predictive influence of each factor generated by SPEAR, we iteratively trained multinomial Lasso classifiers using the *glmnet* R-package with increasing numbers of SPEAR multi-omics factors used as the predictive features. The multi-omics samples were divided into the same training and testing cohorts as described in the SPEAR manuscript. We inspected the balanced misclassification error rate on the training and testing cohorts (Supplementary Fig. 5a, Supplementary Fig. 6a) as well as the coefficient magnitudes for each SPEAR factor for each Lasso model (Supplementary Fig. 5b, Supplementary Fig. 6b).

We found that while Lasso models with fewer SPEAR factors were able to predict well in each dataset, best performance for the prediction of many response classes was found using combinations of many SPEAR factors, suggesting that a singular phenotypes may be driven by multiple complex underlying biological signals.

Supplementary Figure 5: TCGA-BC Factor Prediction

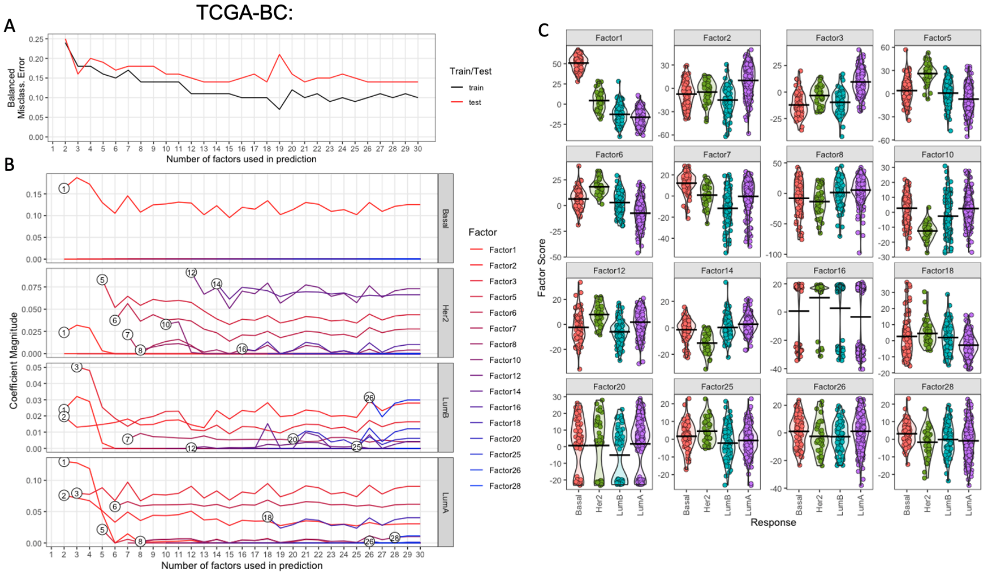

(A) Balanced misclassification error of training and test sets using combinations of SPEAR factors from the TCGA-BC dataset in a multinomial Lasso classifier to predict tumor subtype (Basal, Her2, LumB, LumA). (B) Coefficient magnitudes of different SPEAR factors in the multinomial Lasso classifier for each combination of factors and separated by tumor subtype. (C) Factor scores for all SPEAR factors found to be significant for the prediction of at least one tumor subtype (magnitude of coefficient ≥ 0.01).

Supplementary Figure 6: COVID-19 Factor Prediction

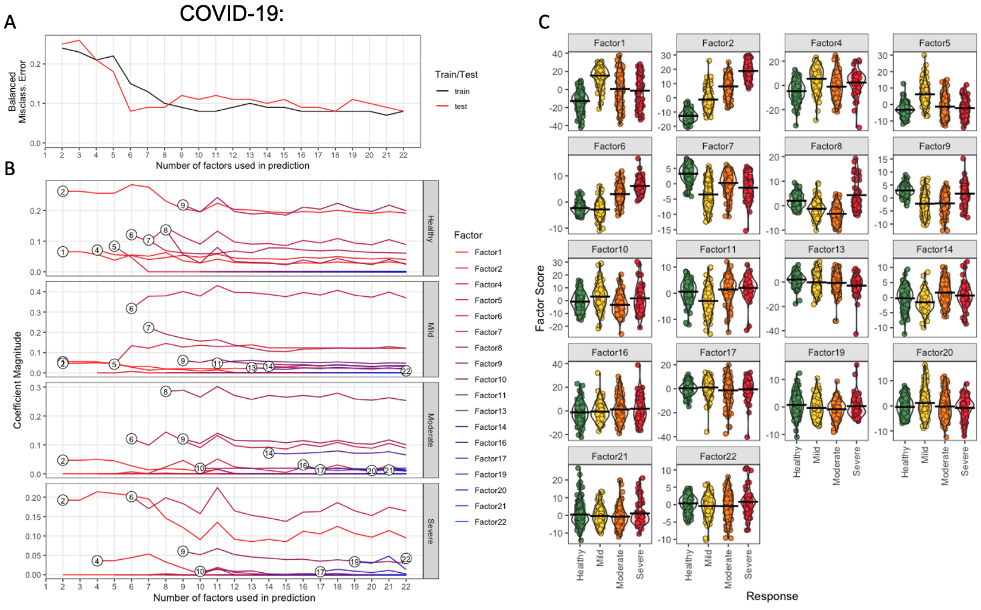

(A) Balanced misclassification error of training and test sets using combinations of SPEAR factors from the COVID-19 dataset in a multinomial Lasso classifier to predict COVID-19 severity (Healthy, Mild, Moderate, Severe). (B) Coefficient magnitudes of different SPEAR factors in the multinomial Lasso classifier for each combination of factors and separated by COVID-19 severity class. (C) Factor scores for all SPEAR factors found to be significant for the prediction of at least one class (magnitude of coefficient ≥ 0.01).

References

Guillaume Bouchard. Efficient bounds for the softmax function, applications to inference in hybrid models. In *Presentation at the Workshop for Approximate Bayesian Inference in Continuous/Hybrid Systems at NIPS-07*. Citeseer, 2007.

Srikantan Nagarajan David Wipf. A new view of automatic relevance determination. *Advances in Neural Information Processing Systems*, 20, 2007.

A. Goldhirsch, E. P. Winer, A. S. Coates, R. D. Gelber, M. Piccart-Gebhart, B. Thürlimann, H.-J. Senn, and Panel members. Personalizing the treatment of women with early breast cancer: highlights of the st gallen international expert consensus on the primary therapy of early breast cancer 2013. 24(9):2206–2223. ISSN 1569-8041. doi: 10.1093/annonc/mdt303.

Michael I Jordan, Zoubin Ghahramani, Tommi S Jaakkola, and Lawrence K Saul. An introduction to variational methods for graphical models. *Machine learning*, 37(2): 183–233, 1999.

Amrit Singh, Casey P. Shannon, Benoît Gautier, Florian Rohart, Michaël Vacher, Scott J. Tebbutt, and Kim-Anh Lê Cao. DIABLO: an integrative approach for identifying key molecular drivers from multi-omics assays. 35(17):3055–3062. ISSN 1367-4811. doi: 10.1093/bioinformatics/bty1054.

Daniel J. Stekhoven and Peter Bühlmann. MissForest—non-parametric missing value imputation for mixed-type data. 28(1):112–118. ISSN 1367-4803. doi: 10.1093/bioinformatics/btr597. URL https://doi.org/10.1093/bioinformatics/btr597.

Yapeng Su, Daniel Chen, Dan Yuan, Christopher Lausted, Jongchan Choi, Chengzhen L. Dai, Valentin Voillet, Venkata R. Duvvuri, Kelsey Scherler, Pamela Troisch, Priyanka Baloni, Guangrong Qin, Brett Smith, Sergey A. Kornilov, Clifford Rostomily, Alex Xu, Jing Li, Shen Dong, Alissa Rothchild, Jing Zhou, Kim Murray, Rick Edmark, Sunga Hong, John E. Heath, John Earls, Rongyu Zhang, Jingyi Xie, Sarah Li, Ryan Roper, Lesley Jones, Yong Zhou, Lee Rowen, Rachel Liu, Sean Mackay, D. Shane O'Mahony, Christopher R. Dale, Julie A. Wallick, Heather A. Algren, Michael A. Zager, Wei Wei, Nathan D. Price, Sui Huang, Naeha Subramanian, Kai Wang, Andrew T. Magis, Jenn J. Hadlock, Leroy Hood, Alan Aderem, Jeffrey A. Bluestone, Lewis L. Lanier, Philip D. Greenberg, Raphael Gottardo, Mark M. Davis, Jason D. Goldman, and James R. Heath. Multi-omics resolves a sharp disease-state shift between mild and moderate COVID-19. 183(6):1479–1495.e20. ISSN 0092-8674. doi: 10.1016/j.cell.2020.10.037. URL https://www.sciencedirect.com/science/article/pii/S0092867420314446.

Brett Wallden, James Storhoff, Torsten Nielsen, Naeem Dowidar, Carl Schaper, Sean Ferree, Shuzhen Liu, Samuel Leung, Gary Geiss, Jacqueline Snider, Tammi Vickery, Sherri R. Davies, Elaine R. Mardis, Michael Gnant, Ivana Sestak, Matthew J. Ellis, Charles M. Perou, Philip S. Bernard, and Joel S. Parker. Development and verification of the PAM50-based prosigna breast cancer gene signature assay. 8(1):54. ISSN 1755-8794. doi: 10.1186/s12920-015-0129-6. URL https://doi.org/10.1186/s12920-015-0129-6.
